# New Pesticidal Diterpenoids from *Vellozia gigantea* (Velloziaceae), an Endemic Neotropical Plant Living in the Endangered Brazilian Biome Rupestrian Grasslands

**DOI:** 10.3390/molecules22010175

**Published:** 2017-01-21

**Authors:** Mariana C. Ferreira, Charles L. Cantrell, Stephen O. Duke, Abbas Ali, Luiz H. Rosa

**Affiliations:** 1 Department of Microbiology, Federal University of Minas Gerais, Belo Horizonte, MG 31270-901, Brazil; marianacostaferreira@yahoo.com.br (M.C.F.); lhrosa@icb.ufmg.br (L.H.R.); 2 United States Department of Agriculture, Agricultural Research Service, Natural Products Utilization Research University, MS 38677, USA; sduke@olemiss.edu; 3 National Center for Natural Products Research, University of Mississippi, MS 38677, USA; aali@olemiss.edu

**Keywords:** diterpene, herbicide, natural products, Velloziacea

## Abstract

*Vellozia gigantea* is a rare, ancient, and endemic neotropical plant present in the Brazilian Rupestrian grasslands. The dichloromethane extract of *V. gigantea* adventitious roots was phytotoxic against *Lactuca sativa*, *Agrostis stolonifera*, and *Lemna paucicostata*, and showed larvicidal activity against *Aedes aegypti*. Phytotoxicity bioassay-directed fractionation of the extract revealed one new isopimaradiene, 8(9),15-isopimaradien-1,3,7,11-tetraone, and three new cleistanthane diterpenoids, 7-oxo-8,11,13-cleistanthatrien-3-ol, 3,20-epoxy-7-oxo-8,11,13-cleistanthatrien-3-ol, and 20-nor-3,7-dioxo-1,8,11,13-cleistanthatetraen-10-ol. These new structures are proposed based on interpretation of ^1^H, ^13^C, COSY, NOESY, HSQC, and HMBC NMR data. 8(9),15-isopimaradien-1,3,7,11-tetraone was especially phytotoxic with an IC_50_ value (30 μM) comparable to those of commercial herbicides clomazone, EPTC, and naptalam. In addition, 7-oxo-8,11,13-cleistanthatrien-3-ol provided 100% mortality at a concentration of 125 ppm against one-day-old *Ae. aegypti* larvae. Our results show that ancient and unique plants, like the endangered narrowly endemic neotropical species *V. gigantea* present in the Rupestrian grasslands, should also be protected because they can be sources of new bioactive compounds.

## 1. Introduction

Brazil has a rich biodiversity of plant species, which includes rare and endemic taxa. Among the typical biomes from Brazil, the Espinhaço mountain range has the Rupestrian grasslands (“campo rupestre”) that shelter different plant species with a high richness of endemism [1,2,3]. The Espinhaço mountain range is located in eastern Brazil (in the states of Minas Gerais and Bahia), extending approximately 1000 km in a north–south direction, and is part of the Biosphere Reserve [4]. The diversity of plant lineages present in the Rupestrian grasslands occurred before the diversification of lowland Brazilian savanna (“cerrado”), suggesting that it may be the most ancient open vegetation in eastern South America [5]. In addition, Brazilian Rupestrian grasslands comprise more than 5000 plant species (about 15% of Brazilian plant diversity), in an area corresponding to 0.78% of its national territory.

Among the endemic plants of the Espinhaço mountain range, the members of the family Velloziaceae occur with high frequency. About 240 predominantly neotropical species of Velloziaceae occur in the eastern mountains of Brazil, especially in the Rupestrian grasslands [1,6]. *Vellozia gigantea* N. L. Menezes & Mello-Silva, known as “canela de ema”, is a rupicolous monocot species that is at least 6 m tall and endemic to the Rupestrian grasslands in Brazil, specifically present in the Serra do Cipó National Park in Minas Gerais state [6]. There are only nine known populations in the region, and they are considered endangered because of disturbance and habitat loss from anthropogenic causes [7].

There is a great need for discovery of natural product-based biopesticides [8]. In the present study, we focus on the chemical fractionation of the adventitious root extract of *V. gigantea* to isolate pure compounds with herbicidal and larvicidal activity.

## 2. Results

### 2.1. Bioassay-Guided Fractionation

**Figure 1:**
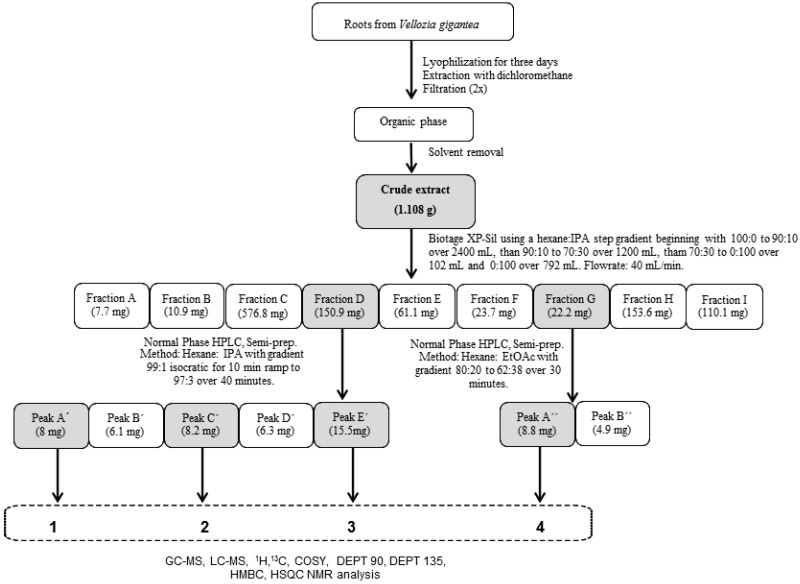
Flowchart illustrating the process of chemical isolation of four phytotoxic compounds produced by *Vellozia gigantea*, which was guided by phytotoxicity bioassays. All bioactive fractions and compounds are shaded.

In a preliminary study, the adventitious roots of *Vellozia gigantea* were extracted using DCM, providing an extract that was subjected to phytotoxicity screening against *L. sativa* and *A. stolonifera*. The crude extract was phytotoxic at 1 mg·mL^−1^ with effect 2 and 4, respectively. The phytotoxic bioassay-directed fractionation of the DCM extract was performed (Figure 1). The fractions A to I obtained from the fractionation of adventitious roots extract were submitted to phytotoxicity assay and fractions D and G displayed activity. Fraction D was phytotoxic at 1 mg·mL^−1^ with effect 2 (*L. sativa*) and 4 (*A. stolonifera*) and fraction G showed the highest phytotoxicity activities at 1 mg·mL^−1^ with effect 4 and 5, respectively. The other fractions showed no significant activity. Based on these results, fractions D and G were selected for isolation of the phytotoxic compounds. From fraction D we isolated three new pure compounds: **1**, **2** and **3**. In addition, from the fraction G, compound **4** was obtained. NMR, GC-MS, and LC-MS analysis, as well as the utilization of search features within the Dictionary of Natural Products on DVD (Version 25:1) and SciFinder, afforded the identification of the compounds as described below.

### 2.2. Identification of Phytotoxic Compounds

**Figure 2:**
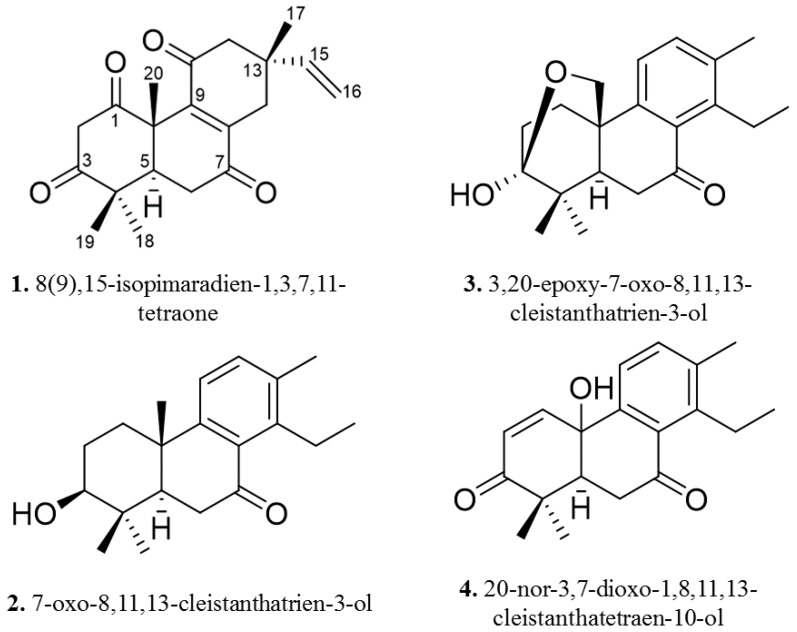
Chemical structures for compounds **1**–**4**.

Peak A′ from HPLC purification of fraction D provided compound **1**, which was identified as 8(9),15-isopimaradien-1,3,7,11-tetraone (**1**) on the basis of its spectroscopic data. Its molecular formula was C_20_H_24_O_4_, as revealed by the pseudomolecular ion at *m*/*z* 327.1597 [M − H]^−^ acquired in the negative ion mode. The presence of four methyl singlets (δ 1.61, 1.26, 1.12, and 1.07) a two-proton olefinic signal at δ 5.04 and an olefinic signal at δ 5.79 in the ^1^H-NMR spectrum combined with olefinic carbons at δ 147.11, 144.18, and 143.28 in the ^13^C-NMR spectrum indicated compound **1** possesses an isopimaradiene skeleton. Four ^13^C-NMR spectral shifts at δ 208.98, 202.51, 199.69, and 198.87 suggested a tetraone configuration. Inspection of the literature revealed similar isopimaradiene diones [9]. Positioning of C-1, C-3, C-7, and C-11 ketones was established by HSQC, HMBC, and COSY spectroscopic data (Supplementary Materials). NOESY correlations established the relative configuration as drawn in Figure 2.

Peak C′ from the HPLC purification of fraction D provided compound **2**, which was identified as 7-oxo-8,11,13-cleistanthatrien-3-ol (**2**) on the basis of its spectroscopic data. Its molecular formula was C_20_H_28_O_2_, as revealed by the pseudomolecular ion at *m*/*z* 301.2173 [M + H]^+^ acquired in positive ion mode. The presence of a methyl triplet (δ 1.19), four methyl singlets (δ 2.31, 1.14, 1.04, and 1.01), and two aromatic proton doublets (δ 7.24, 7.05) in the ^1^H-NMR spectrum combined with information from the ^13^C-NMR spectrum (six sp^2^ hybridized olefinic carbons at δ 153.89, 144.38, 135.60, 134.83, 131.00, and 120.68) indicated that compound **2** possesses a cleistanthatriene skeleton. Inspection of the literature indicated similarities to 7-oxo-8,11,13-cleistanthatriene with differences in the A ring carbon shifts [10]. In particular, compound **2** had an additional sp^3^ oxygenated carbon at δ 78.44 and an oxymethine proton at δ 3.31. Positioning at C-3 was established by HSQC, HMBC, and COSY spectroscopic data (Supplementary Materials). NOESY correlations between H-3 and H-5 as well as between H-20 and H-19 established the relative configuration as drawn in Figure 2.

Peak E′ from the HPLC purification of fraction D provided compound **3**, which was identified as 3,20-epoxy-7-oxo-8,11,13-cleistanthatrien-3-ol on the basis of its spectroscopic data. Its molecular formula was C_20_H_26_O_3_, as revealed by the pseudomolecular ion at *m*/*z* 337.1797 [M + Na]^+^ acquired in positive ion mode. The presence of a methyl triplet (δ 1.15), three methyl singlets (δ 2.30, 1.10, 1.02), and two aromatic proton doublets (δ 7.25, 7.09) in the ^1^H-NMR spectrum combined with information from the ^13^C-NMR spectrum (six sp^2^ hybridized olefinic carbons at δ 144.28, 142.96, 136.48, 134.83, 131.80, 124.40) indicated that compound **3** possesses a cleistanthatriene skeleton. COSY, HSQC, and HMBC data established the positions of the C-7 ketone and the hydroxy carbon at C-20. Analysis of the degree of unsaturation for **3** indicated the presence of an additional ring and a hemiketal methine carbon at δ 99.02. HMBC correlations between the two oxymethylene protons at δ 4.21 (1H, dd, *J* = 9.5, 2.9 Hz, H-20a) and δ 3.99 (1H, d, *J* = 9.4 Hz, H-20b) and the C-3 hemiketal carbon firmly established the location of the additional ring in the form of an epoxide between C-3 and C-20. Such epoxides in the cleistanthatriene skeleton have been reported previously [11]. NOESY spectrum analysis indicated correlations between oxymethines at C-20 and the beta methyl at C-19 as well as the C-6 beta methylene proton at δ 2.51. This and other correlations helped establish the relative configuration for **3** and establish it as a new compound.

Peak A″ from the HPLC purification of fraction G provided compound **4**, which was identified as 20-nor-3,7-oxo-1,8,11,13-cleistanthatetraen-10-ol on the basis of its spectroscopic data. Its molecular formula was C_19_H_22_O_3_, as revealed by the pseudomolecular ion at *m*/*z* 299.1669 [M + H]^+^ acquired in positive ion mode. The presence of a methyl triplet (δ 1.16), three methyl singlets (δ 2.38, 1.24, 0.63), and two aromatic proton doublets (δ 7.24, 7.34) in the ^1^H-NMR spectrum combined with information from the ^13^C-NMR spectrum (eight sp^2^ hybridized olefinic carbons at δ 150.04, 144.46, 143.48, 138.76, 135.00, 131.75, 127.44 and 123.41) indicated that compound **4** possesses a cleistanthatetraene skeleton. C-3 and C-7 ketone positions were easily established using HMBC correlation data. Positioning of the hydroxyl at C-10 was unequivocally confirmed with HMBC correlations between H-11 and C-10, among others. NOESY correlation data was unable to establish the relative configuration of the hydroxyl at C-10 due to the lack of definitive correlation data. Complete structure determination was accomplished using COSY, HSQC, HMBC, and DEPT spectroscopic data (Supplementary Materials) unequivocally establishing the structure as drawn in Figure 2.

### 2.3. Phytotoxic Activity of the Pure Compounds

**Figure 3:**
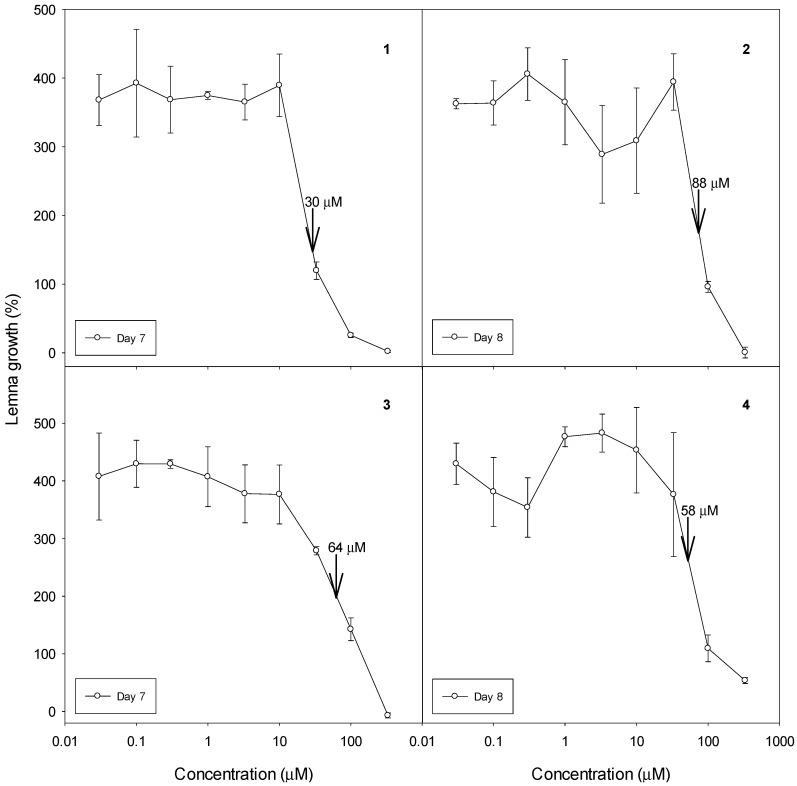
Dose/response curves for the four isolated compounds in a *L. paucicostata* bioassay. Error bars are ±SE of the mean. Arrows with numbers denote the concentration that inhibits growth by 50%. (**1**) 8(9),15-isopimaradien-1,3,7,11-tetraone; (**2**) 7-oxo-8,11,13-cleistanthatrien-3-ol; (**3**) 3,20-epoxy-7-oxo-8,11,13-cleistanthatrien-3-ol; (**4**) 20-nor-3,7-dioxo-1,8,11,13-cleistanthatetraen-10-ol.

The phytotoxic activities of the pure compounds against *L. sativa* and *A. stolonifera* are shown in Table 1. At 1000 µg·mL^−1^, compounds 8(9),15-isopimaradien-1,3,7,11-tetraone (**1**), 7-oxo-8,11,13-cleistanthatrien-3-ol (**2**) and 20-nor-3,7-dioxo-1,8,11,13-cleistanthatetraen-10-ol (**4**) were moderately active (2–4 ranking) on both species, whereas 3,20-epoxy-7-oxo-8,11,13-cleistanthatrien-3-ol (**3**) had no activity on lettuce at this concentration, but was moderately active on *A. stolonifera*. We can observe that after the fractionation of fraction G to obtain compound **4**, there was a reduction of the activity initially expressed. This fact may suggest that the initial activity of fraction G (4/5 effect) could be related not only to compound **4** (showed 2/3 effect), but may also be acting synergistically between this compound and some other compound present in the fraction. Additionally, all four compounds were active as growth inhibitors of *L. paucicostata*, with the order of activity being **1** > **4** > **3** > **2** (Figure 3). With all four compounds, there was no significant effect at 10 μM and complete cessation of growth at 333 μM, except for **4**, for which there was almost complete growth inhibition. The IC_50_ values are comparable to the commercial herbicides clomazone (126 μM), EPTC (62 μM), and naptalam (128 μM) in the same bioassay [12].

### 2.4. Larval Bioassays

In larvicide bioassays of the pure compounds against *Aedes aegypti*, 7-oxo-8,11,13-cleistanthatrien-3-ol (**2**) was most toxic against one-day-old *Ae. aegypti* larvae at 24 h post-treatment, with 100% mortality at the highest concentration of 125 ppm, followed by 40% and 10% at 62.5 and 31.25 ppm, respectively. Additionally, 3,20-epoxy-7-oxo-8,11,13-cleistanthatrien-3-ol (**3**) caused 50% mortality at all the above dosages. The compounds 20-nor-3,7-dioxo-1,8,11,13-cleistanthatetraen-10-ol (**4**) and 8(9),15-isopimaradien-1,3,7,11-tetraone (**1**) had no activity against one-day-old *Ae. aegypti* larvae at the highest dose of 125 ppm.

## 3. Materials and Methods

### 3.1. Study Area

Fragments of adventitious roots of 18 specimens of *V. gigantea* were collected, without damage to the natural plant populations, in May 2013, at Serra do Cipó National Park (19°14′874″ S; 043°30′574″ W), a protected area in Minas Gerais state, Brazil. Identification of *V. gigantea* was done by comparisons with the voucher specimen deposited at the herbarium of Institute of Biological Science (BHCB) of the Federal University of Minas Gerais, Brazil under the code BHCB 102620 [13]. Access to the plants was according to the Brazilian biological diversity rules.

### 3.2. Adventitious Roots Extract Production

Fresh adventitious roots of *V. gigantea* of 18 individual plants (approximately 10 g/individual) were dried by lyophilization for three days to remove residual water. The lyophylized adventitious root material was extracted in a beaker with 2 L of dichloromethane (DCM, Fisher, Pittsburgh, PA, USA) for five days. The DCM was removed in a rotary evaporator (IKA^®^RV 10) under a partial vacuum at temperatures below 35 °C. The residual solvent was eliminated overnight in a vacuum centrifugal concentrator (Thermo Savant, Pittsburgh, PA, USA) at 35 °C. This process yielded 4 g of crude extract, which was stored at −20 °C until the chemical fractionation guided by phytotoxicity assays could be completed.

### 3.3. Adventitious Roots Extract Fractionation

The adventitious root crude extract fractionation was performed by column chromatography, using the Biotage system, Inc. Isolera™ pump (Charlotte, NC, USA) equipped with an Isolera™ flash collector and variable wavelength detector. Initially, 1.108 g of root crude extract was adsorbed to silica gel and applied to a silica gel chromatography column (40–63 μm, 40 mm × 150 mm, 60 Å), running at 40 mL/min using a hexane/Isopropanol with gradient of 100% hexane—0% IPA to 90% hexane—10% IPA over 2400 mL, them 90% hexane—10% IPA to 70% hexane—30% IPA over 1200 mL, them 70% hexane—30% IPA to 0% hexane—100% IPA over 102 mL and finishing with a 100% IPA for wash over 792 mL. Portions of 27 mL fractions were collected and recombined based on TLC similarities into nine fractions [A (7.7 mg), B (10.9 mg), C (576.8 mg), D (150.9 mg), E (61.1 mg), F (23.7 mg), G (22.2 mg), H (153.6 mg), and I (110.1 mg)].

### 3.4. NMR Spectroscopy

The root crude extract, fractions, and pure compounds were analyzed by NMR spectroscopy on a Varian INOVA 500 MHz spectrometer (Palo Alto, CA, USA). NMR spectra were recorded in CDCl_3_.

### 3.5. HPLC Analysis

Fractions D and G, which are phytotoxic, were purified by HPLC. An Agilent 1200 system equipped with a quaternary pump, autosampler, diode-array detector, and vacuum degasser was used with a Zorbax RX-SIL (Agilent, Santa Clara, CA, USA) 5 µm 9.4 mm × 250 mm HPLC column. A total of 150.9 mg of fraction D was dissolved in methanol and purified. The manual injection for each sample was 20 µL. The method was isocratic 99% hexane: 1% IPA for 10 min ramp to gradient 97% hexane: 3% IPA over 40 min. After running two samples, the column was washed with hexane:IPA for 5 min and re-equilibrated. Analytes were detected at 254 and 280 nm by diode array detector. Five major peaks were detected and collected [A′ (8 mg), B′ (6.1 mg), C′ (8.2 mg), D′ (6.3 mg), and E′ (15.5 mg)]. However, only the A′ (**1**), C′ (**2**), and E′ (**3**) peaks were characterized and identified.

A total of 22 mg of fraction G was dissolved in methanol and purified by normal phase HPLC System. The manual injection for each sample was 20 µL. The method was 80% hexane:20% EtOAc to 62% hexane:38% EtOAc over 30 min. For each running the column was re-equilibrated. Analytes was detected at 254 and 280 nm by diode array detector. Two major peaks were detected and collected [A″ (8.8 mg), B″ (4.9 mg)]. However, only the A″ peak (**4**) was characterized and identified.

*8(9),15-Isopimaradien-1,3,7,11-tetraone* (**1**). High-resolution ESI-MS: *m*/*z* 327.1597 [M − H]^−^; calculated for C_20_H_23_O_4_, 327.1596. [α]${}_{D}^{25}$ = +33.5 (*c* 0.275, CHCl_3_). ^1^H-NMR (CDCl_3_, 500 MHz) δ 5.79 (1H, dd, *J* = 17.5, 10.7 Hz, H-15), 5.04 (2H, dd, *J* = 14.1, 3.2 Hz, H-16), 3.70 (1H, d, *J* = 19.9 Hz, H-2), 3.39 (1H, d, *J* = 19.9 Hz, H-2), 2.76 (1H, d, *J* = 15.7 Hz, H-12), 2.67 (1H, d, *J* = 18.5 Hz, H-14), 2.61–2.54 (2H, m, H-6), 2.40 (1H, d, *J* = 18.5 Hz, H-14), 2.33 (1H, d, *J* = 15.8 Hz, H-12), 2.15 (1H, dd, *J* = 13.3, 5.0 Hz, H-5), 1.61 (3H, s, H-20), 1.26 (3H, s, H-19), 1.12 (3H, s, H-18), 1.07 (3H, s, H-17). ^13^C-NMR (126 MHz, CDCl_3_) δ 208.98 (C-3), 202.51 (C-1), 199.69 (C-11), 198.87 (C-7), 147.11 (C-9), 144.18 (C-15), 143.28 (C-8), 113.06 (C-16), 53.46 (C-10), 50.60 (C-2), 49.68 (C-12), 47.00 (C-4), 45.33 (C-5), 38.08 (C-13), 34.75 (C-14), 34.55 (C-6), 28.52 (C-18), 25.79 (C-17), 21.55 (C-19), 15.59 (C-20).

*7-oxo-8,11,13-Cleistanthatrien-3-ol* (**2**). High-resolution ESI-MS: *m*/*z* 301.2173 [M + H]^+^; calculated for C_20_H_29_O_2_, 301.2168. [α]${}_{D}^{25}$ = +9.7 (*c* 0.33, CHCl_3_). ^1^H-NMR (CDCl_3_, 500 MHz) δ 7.24 (1H, d, *J* = 8.0 Hz, H-12), 7.05 (1H, d, *J* = 8.0 Hz, H-11), 3.31 (1H, dd, *J* = 10.8, 4.5 Hz, H-3), 3.00–2.89 (1H, m, H15a), 2.87–2.79 (1H, m, H-15b), 2.72–2.66 (2H, m, H-6), 2.31 (3H, s, H-17), 2.26 (1H, dt, *J* = 12.7, 3.0 Hz, H-1a), 1.88 (1H, m, H-2), 1.83 (1H, m, H-5), 1.75 (1H, m, H-1b), 1.19 (3H, t, *J* = 7.4 Hz, H-16), 1.14 (3H, s, H-20), 1.04 (3H, s, H-18), 1.01 (3H, s, H-19). ^13^C-NMR (126 MHz, CDCl_3_) δ 201.83 (C-7), 153.89 (C-9), 144.38 (C-14), 135.60 (C-13), 134.83 (C-12), 131.00 (C-8), 120.68 (C-11), 78.44 (C-3), 47.32 (C-5), 39.04 (C-4), 38.11 (C-6), 37.98 (C-10), 36.77 (C-1), 27.74 (C-2), 27.39 (C-18), 23.71 (C-15), 23.36 (C-20), 19.29 (C-17), 14.91 (C-19), 14.64 (C-16).

*3,20-Epoxy-7-oxo-8,11,13-cleistanthatrien-3-ol* (**3**). High-resolution ESI-MS: *m*/*z* 337.1797 [M + Na]^+^; calculated for C_20_H_26_O_3_Na, 337.1779. [α]${}_{D}^{25}$ = +28.9 (*c* 0.56, CHCl_3_). ^1^H-NMR (CDCl_3_, 500 MHz) δ 7.25 (1H, d, *J* = 7.9 Hz, H-12), 7.09 (1H, d, *J* = 8.2 Hz, H-11), 4.21 (1H, dd, *J* = 9.5, 2.9 Hz, H-20a), 3.99 (1H, d, *J* = 9.4 Hz, H-20b), 3.02–2.82 (2H, m, H-15), 2.78 (1H, dd, *J* = 15.0, 13.2 Hz, H-6a), 2.51 (1H, dd, *J* = 13.1, 3.5 Hz, H-6b), 2.49–2.42 (1H, m, H-1a), 2.30 (3H, s, H-17), 2.25 (1H, ddd, *J* = 13.4, 11.7, 5.5 Hz, H-2), 2.20–2.11 (1H, m, H-5), 1.94 (1H, ddd, *J* = 13.4, 11.8, 3.7 Hz, H-2b), 1.15 (3H, t, *J* = 7.4 Hz, H-16), 1.10 (3H, s, H-18), 1.02 (3H, s, H-19). ^13^C-NMR (126 MHz, CDCl_3_) δ 201.87 (C-7), 144.28 (C-14), 142.96 (C-9), 136.48 (C-13), 134.83 (C-12), 131.80 (C-8), 124.40 (C-11), 99.02 (C-3), 71.14 (C-20), 47.30 (C-5), 40.55 (C-4), 39.27 (C-6), 37.11 (C-10), 34.80 (C-1), 29.46 (C-2), 26.76 (C-19), 23.52 (C-15), 19.42 (C-17), 18.14 (C-18), 14.51 (C-16).

*20-nor-3,7-dioxo-1,8,11,13-Cleistanthatetraen-10-ol* (**4**). High-resolution ESI-MS: *m*/*z* 299.1669 [M + H]^+^; calculated for C_19_H_23_O_3_, 299.1647. [α]${}_{D}^{25}$ = −216.9 (*c* 0.065, CHCl_3_). ^1^H-NMR (CDCl_3_, 500 MHz) δ 7.34 (1H, d, *J* = 7.9 Hz, H-12), 7.24 (1H, d, *J* = 8.0 Hz, H-11), 7.17 (1H, d, *J* = 10.3 Hz, H-1), 6.07 (1H, d, *J* = 10.2 Hz, H-2), 3.09 (1H, m, H-6a), 3.07 (1H, m, H-15a), 2.86 (1H, m, H-15b), 2.73 (1H, dd, *J* = 7.6, 4.8 Hz, H-5), 2.50 (1H, dd, *J* = 16.8, 4.9 Hz, H-6b), 2.38 (3H, s, H-17), 1.24 (3H, s, H-19), 1.16 (3H, t, *J* = 7.4 Hz, H-16), 0.63 (3H, s, H-18). ^13^C-NMR (126 MHz, CDCl_3_) δ 203.12 (C-3), 199.77 (C-7), 150.04 (C-1), 144.46 (C-14), 143.48 (C-9), 138.76 (C-13), 135.00 (C-12), 131.75 (C-8), 127.44 (C-2), 123.41 (C-11), 70.17 (C-10), 49.93 (C-5), 45.54 (C-4), 39.09 (C-6), 24.67 (C-19), 23.11 (C-15), 21.28 (C-18), 19.61 (C-17), 14.47 (C-16).

### 3.6. High-Resolution LC-MS Analysis

High-resolution mass spectra (ESI-MS) were obtained using an Agilent 1100 HPLC coupled to a JEOL AccuTOF (JMS-T100LC) (Peabody, MA, USA). All isolated compounds were prepared in MeOH and injected directly into a 0.3 mL/min stream of either MeOH or 80% MeOH/20% DI H_2_0. Twenty microliters of sample (approximately 0.1 mg/mL) were injected manually at 0.5 min while mass drift compensation standards [*L*-tryptophan (negative ion), PEG (positive ion)] were injected at 1.5 min over the course of a 2 min run.

### 3.7. Bioassays with Lettuce and Bentgrass

Seeds of lettuce (*Lactuca sativa—*Iceberg A Crisphead cultivar from Burpee Seeds, Warminster, PA, USA) and bentgrass (*Agrostis stolonifera—*Penncross variety obtained from Turf-Seed, Inc. of Hubbard, OR, USA) were surface sterilized with a 0.5% to 1% (*v*/*v*) sodium hypochlorite solution for approximately 10 min, rinsed with deionized water and dried in a sterile environment. The DCM crude extract of the adventitious roots of *V. gigantea*, column chromatography fractions, and pure compounds were tested for phytotoxicity on the monocot bentgrass and dicot lettuce according to published methods [14]. A filter paper disk (Whatman Grade 1, 1.5 cm) was placed in each well of a 24-well plate. The control wells contained 200 μL of deionized water. The control + solvent well contained 180 μL of water and 20 μL of the solvent. All sample wells contained 180 μL of water and 20 μL of the appropriate dilution of the sample. Water was pipetted into the well before the sample or solvent. Test samples were dissolved in acetone and the final concentration of acetone in the wells was 10%. For the bioassay five lettuce seeds or 10 mg of bentgrass seeds were placed in each well before sealing the plate with Parafilm. The plates were incubated either for five days (lettuce) or 12 days (bentgrass) in a Percival Scientific (Perry, IA, USA) CU-36L5 incubator under continuous light conditions at 26 °C and 120 μmol∙s^−1^∙m^−2^ average photosynthetically active radiation (PAR). A qualitative estimate of phytotoxicity was made by assigning a rating of 0 for no effect (sample well plants looked identical to the control + solvent well plants; seeds had germinated and resulting seedlings had grown normally), 2 for less than 50% germination inhibition, 3 for about 50% germination inhibition, 4 for more than 50% germination inhibition, and 5 for no germination of the seeds. Each experiment was repeated three times.

### 3.8. Bioassay with Lemna Paucicostata (Duckweed)

The bioassay with *Lemna paucicostata* (duckweed) was carried out using the method of Michel et al [12]. Duckweed stocks were grown from a single colony consisting of a mother and two daughter fronds in 100 mL of Hoagland’s No. 2 Basal Salt Mixture (Sigma H2395) (1.6 g∙L^−1^) with added iron (1 mL of 1000× Fe-EDTA solution to 1 L of Hoagland media, pH adjusted to 5.5) in sterile jars with vented lids in a Percival Scientific CU-36L5 incubator under continuous light conditions at 26 °C and 120 μmol∙s^−1^∙m^−2^ average PAR. The iron solution (1000×) contained 18.36 g∙L^−1^ of Fe-EDTA. Media were changed every two to three days or new stocks were prepared in fresh media. Plant doubling time was approximately 24 to 36 hours. Both screening and replicate series tests were conducted using non-pyrogenic polystyrene and sterile six-well plates (CoStar 3506, Corning Incorporated). Each well contained 4950 μL of the Hoagland’s media plus 50 μL of water, or the solvent, or the compound dissolved in the appropriate solvent (at a concentration of 100×). The final concentration of the solvent was therefore approximately 1% by volume. A graphic template of the six-well plates was used for LemnaTec (LemnaTec, Würselen, Germany) image analysis software. Each well was inoculated with two three-frond plants of the same age (four to five days old) and approximate size and incubated in the Percival incubator as described above. Each treatment was replicated three times. IC_50_ (concentration inhibiting growth by 50%) values were determined with R statistics software [15]. Plant frond areas were measured at time intervals (0–7 days) using a LemnaTec image analysis methodology that records frond number, total frond area, as well as color classes that indicate chlorotic or necrotic effect.

### 3.9. Mosquito Larvae Bioassays

Bioassays were conducted by using the bioassay system described by Ali et al. [16] to determine the larvicidal activity of the pure compounds isolated from *V. gigantea* against *Aedes aegypti*. Five one-day-old larvae were transferred to each well of 24-well tissue culture plates in a 30–40 µL droplet of water. An aliquot of 50 µL of larval diet (2% slurry of 3:2 Beef Liver powder and Brewer’s yeast and 1 mL of deionized water were added to each well using a Finnpipette stepper (Thermo Fisher, Vantaa, Finland). All the compounds were diluted in dimethyl sulfoxide (DMSO). Larval mortality was recorded 24 h post-treatment. Larvae that showed no movement in the well after manual disturbance were recorded as dead. Larval mortality was converted into a percentage.

## 4. Discussion

*Vellozia gigantea* is a unique and endemic species living only in the Rupestrian grasslands biome in Brazil. From the extract of adventitious roots of *V. gigantea*, we isolated four new diterpenes with phytotoxic and larvicidal activity. Other diterpenes from plants have been found to have pesticidal activities in the past [17,18,19]. Other diterpenes have been reported from the roots of other Velloziacea species: diterpenes with the isopimarane skeleton from *V. bilocor* [20], pimarane-type from *V.* flavicans [21] and 19-hydroxy-8(9),15-abietadiene, 19-hydroxy-8(9), 13(16)-14*S*,17-cyclolabdadiene, and 17-hydroxy-8(9),15-isopimaradien from *V. compacta* [22]. Few compounds have been described for *V. gigantea* until now. Morales et al. [23] described the antioxidant tocotrienols (tocopherols and tocotrienols) accumulated in the leaves of *V. gigantea*.

In conclusion, our study proposes the structures of four new natural diterpene compounds produced by the adventitious root tissues of *V. gigantea*. These compounds might be used as scaffolds to develop new herbicides and other pesticides. Additionally, our results show that ancient and unique plants, like the endangered, narrowly endemic neotropical species *V. gigantea* present in the Rupestrian grasslands, should be protected because of their intrinsic value as sources of new, bioactive compounds. As *V. gigantea* is an endangered species, further studies will be necessary to obtain micropropagated plants and isolate these new pesticidal diterpenes, which may be modified to increase their activity and modify their selectivity.

## Figures and Tables

**Table 1:** Herbicidal activity of pure compounds isolated from adventitious roots of *Vellozia gigantean*.

Compounds	Tested Concentration (µg·mL^−1^)	Phytotoxicity ^a^	
		*Lactuca sativa*	*Agrostis stolonifera*
**1**	1	0	0
	10	0	0
	100	1	0
	1000	2	3
**2**	1	0	0
	10	0	0
	100	2	1
	1000	3	4
**3**	1	0	0
	10	0	0
	100	0	0
	1000	0	3
**4**	1	0	0
	10	0	0
	100	0	0
	1000	2	3

^a^ The qualitative estimate of phytotoxicity was evaluated using a rating scale of 0–5, where 0 = no effect and 5 = no growth or no germination of the seeds.

## Supplementary Materials

Supplementary materials can be accessed at: http://www.mdpi.com/1420-3049/22/1/175/s1.

## References

[1] Lousada J.M., Borba E.L., Ribeiro K.T., Ribeiro L.C., Lovato M.B. (2011). Genetic structure and variability of the endemic and vulnerable *Vellozia gigantea* (*Velloziaceae*) associated with the landscape in the Espinhaço Range, in southeastern Brazil: implications for conservation. Genetica.

[2] Giulietti A.M., Pirani J.R., Vanzolini P.E., Heyer W.R. (1988). Patterns of geographic distribution of some plant species from the Espinhaco Range, Minas Gerais and Bahia, Brazil. Proceedings of a Workshop on Neotropical Distribution Patterns.

[3] Joly A.B. (1970). Conheça a Vegetação Brasileira.

[4] United Nations Educational, Scientific and Cultural Organization (UNESCO) MAB Biosphere Reserves Directory. http://www.brasilia.unesco.org/noticias/releases/2005/biosferaespinhaco.

[5] Silveira F.A.O., Negreiros D., Barbosa N.P.U., Buisson E., Carmo F.F., Carstensen D.W., Conceição A.A., Cornelissen T.G., Ecthernacht L., Fernandes G.W. (2016). Ecology and evolution of plant diversity in the endangered campo rupestre: A neglected conservation priority. Plant Soil.

[6] Mello-Silva R., Menezes N.L. (1999). Two new Brazilian Velloziaceae, *Vellozia auriculata* and *Vellozia gigantea*, and a key to the related dracenoid species of *Vellozia*. Novon.

[7] Biodiversitas (2005). Lista da Flora Brasileira Ameaçada de Extinção.

[8] Duke S.O., Owens D.K., Dayan F.E. (2014). The growing need for biochemical bioherbicides. Am. Chem. Soc. Symp. Ser..

[9] Pinto A.C., Valente L.M.M., da Silva R.S. (1988). Norditerpenoids from *Vellozia pusilla*. Phytochemistry.

[10] Pinto A.C., Pereira A.L., Antunes O.A.C. (1985). RMN ^13^C de diterpenoids com esqueleto cleistantano. Quim. Nova.

[11] Cheng L., Ji K., Liao S., Gan L., Yang L., Cao D., Liu Y., Guo J., Zhang P., Lu C. (2016). Diterpenoids and Phenanthrenones from the Leaves and Stems of Strophioblachia Fimbricalyx. Tetrahedron Lett..

[12] Michel A., Johnson R.D., Duke S.O., Scheffler B.E. (2004). Dose-response relationships between herbicides with different modes of action and growth of *Lemna paucicostata:* An improved exotoxicological method. Environ. Toxicol. Chem..

[13] Herbarium of Institute of Biological Science (BHCB) of the Federal University of Minas Gerais, Brazil. http://sciweb.nybg.org/science2/IndexHerbariorum.asp.

[14] Dayan F.E., Romagni J.G., Duke S.O. (2000). Investigating the mode of action of natural phytotoxins. J. Chem. Ecol..

[15] R-Development-Core-Team (2015). R: A Language and Environment for Statistical Computing.

[16] Ali A., Tabanca N., Demirci B., Baser K.H.C., Ellis J., Gray S., Lackey B.R., Murphy C., Khan I.A., Wedge D.E. (2013). Composition, mosquito larvicidal, biting deterrent and antifungal activity of essential oils of different plant parts of *Cupressus arizonica* var. glabra (Sudw.) Little (‘Carolina Sapphire’). Nat. Prod. Commun..

[17] Duke S.O., Romagni J.G., Dayan F.E. (2000). Natural products as sources for new mechanisms of herbicidal action. Crop Protect..

[18] Tellez M.R., Duke S.O., Schrader K.K., Dayan F.E., Romagni J.G., Kobaisy M., Kuo T.M., Gardner H.W. (2002). Terpenoid-based defense in plants and other organisms. Lipid Biotechnology.

[19] Duke S.O., Oliva A., Macías F.A., Galindo J.C.G., Molinillo J.M.G. (2004). Mode of action of phytotoxic terpenoids. Allelopathy: Chemistry and Mode of Action of Allelochemicals.

[20] Pinto A.C., Queiroz P.P.S., Garcez W.S. (1991). Diterpenes from *Vellozia bicolor*. J. Brm. Chem. Soc..

[21] Pinto A.C., Rezende C.M., Antunes O.A., Correia C.R.D. (1996). Three isomeric diterpenes from Vellozia flavicans. Phytochemistry.

[22] Pinto A.C., Borges C. (1983). Six diterpenes from Vellozia compacta. Phytochemistry.

[23] Morales M., Garcia Q.S., Siqueira-Silva A.I., Silva M.C., Munne-Bosch S. (2014). Tocotrienols in *Vellozia gigantea* leaves: Occurrence and modulation by seasonal and plant size effects. Planta.

