# Supplementary material for: New Pesticidal Diterpenoids from Vellozia gigantea (Velloziaceae), an Endemic Neotropical Plant Living in the Endangered Brazilian Biome Rupestrian Grasslands
Source: Molecules. 2017 Jan 21;22(1):175. doi: 10.3390/molecules22010175 (PMC6155815; doi:10.3390/molecules22010175)
Supplement: Supplementary file 1 [file molecules-22-00175-s001.pdf]

Supplementary Materials: New Pesticidal Diterpenoids from *Vellozia gigantea* (Velloziaceae), an Endemic Neotropical Plant Living in the Endangered Brazilian Biome Rupestrian Grasslands

Mariana C. Ferreira, Charles L. Cantrell, Stephen O. Duke, Abbas Ali and Luiz H. Rosa

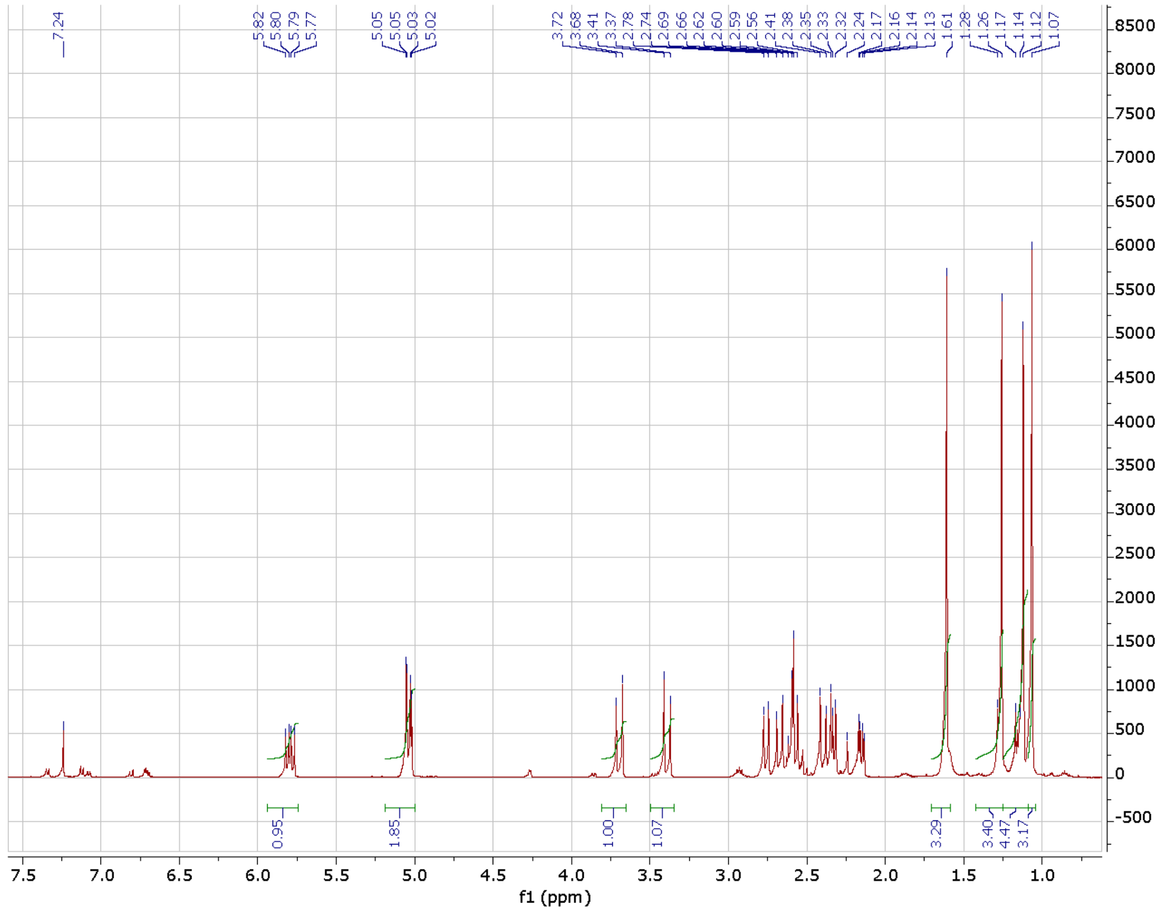

Figure S1. ^{1}H-NMR (500 MHz) Data for 8(9),15-isopimaradien-1,3,7,11-tetraone (**1**).

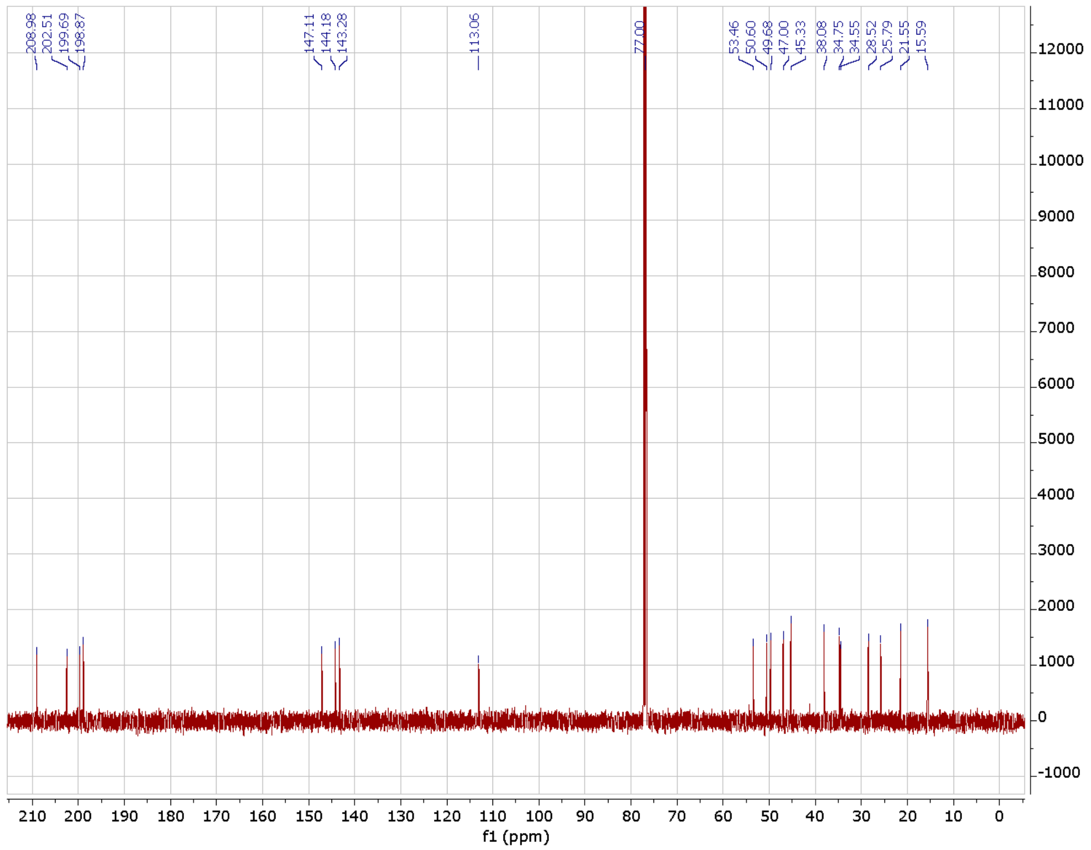

Figure S2. ^{13}C-NMR (125 MHz) Data for 8(9),15-isopimaradien-1,3,7,11-tetraone (**1**).

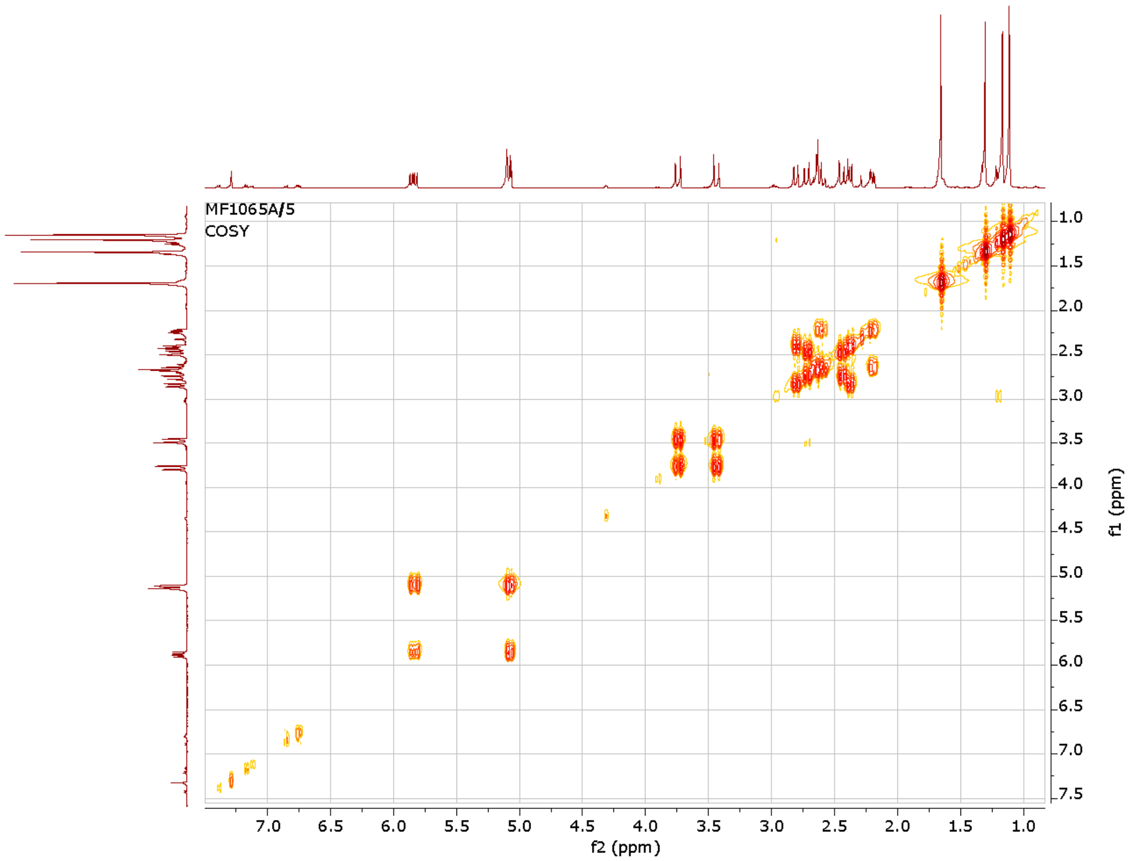

Figure S3. ^{1}H–^{1}H COSY (500 MHz) Data for 8(9),15-isopimaradien-1,3,7,11-tetraone (**1**).

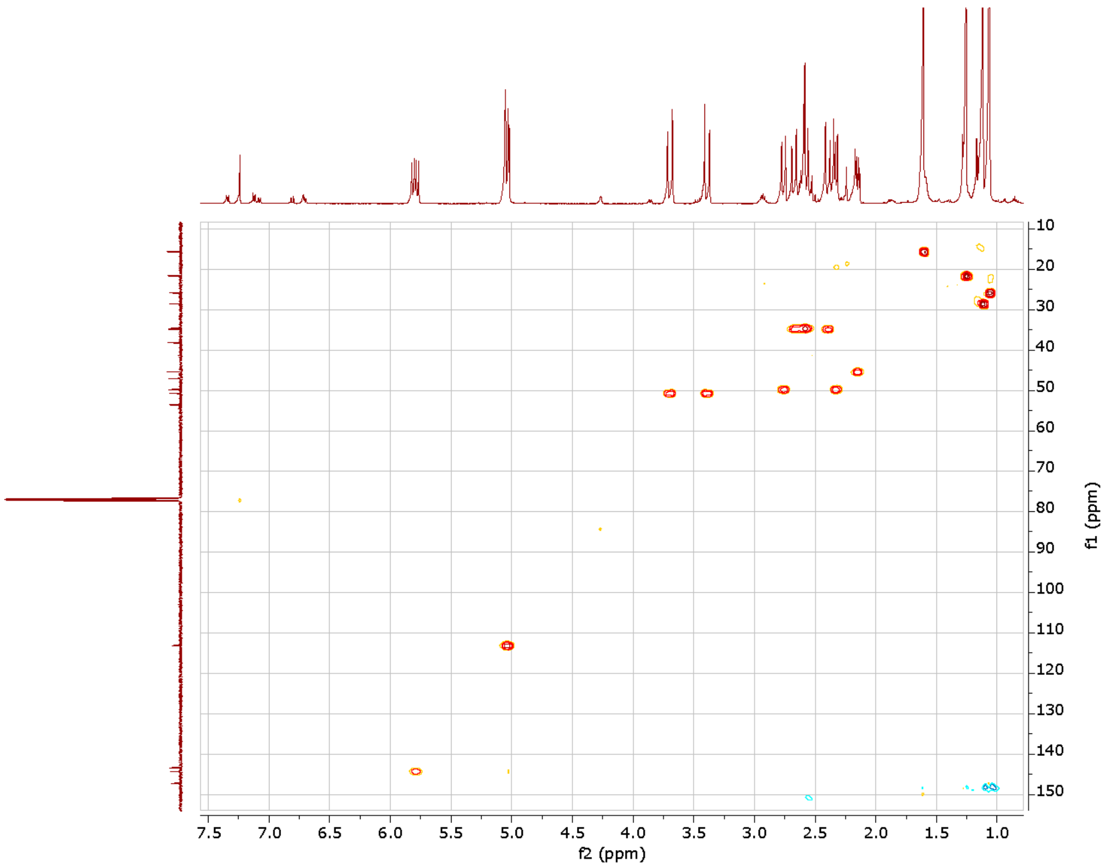

Figure S4. HSQC (500 MHz) Data for 8(9),15-isopimaradien-1,3,7,11-tetraone (**1**).

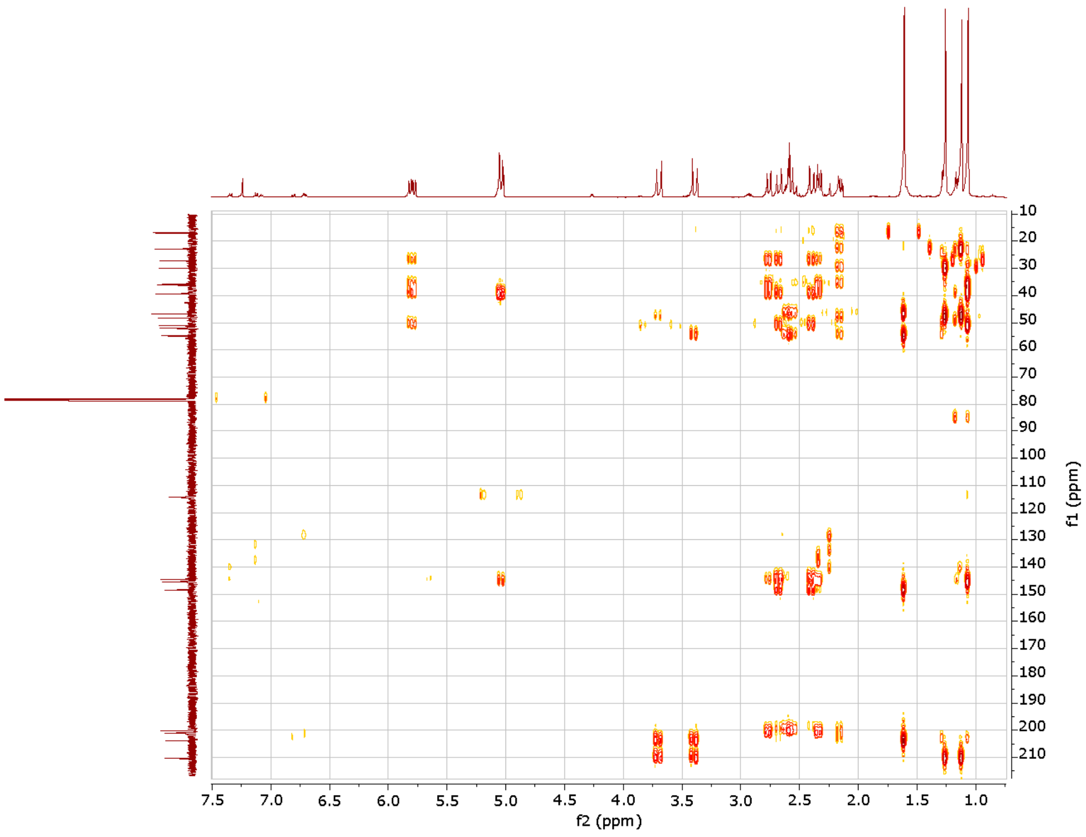

Figure S5. HMBC (500 MHz) Data for 8(9),15-isopimaradien-1,3,7,11-tetraone (**1**).

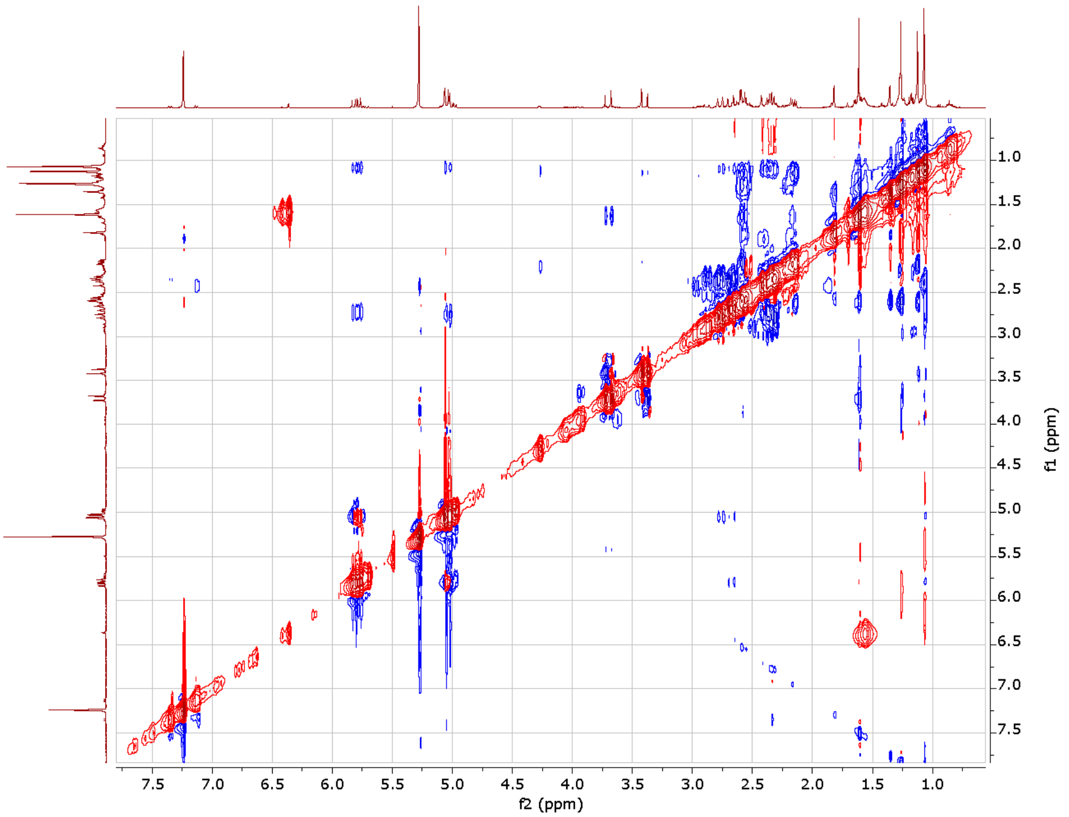

Figure S6. NOESY (500 MHz) Data for 8(9),15-isopimaradien-1,3,7,11-tetraone (**1**).

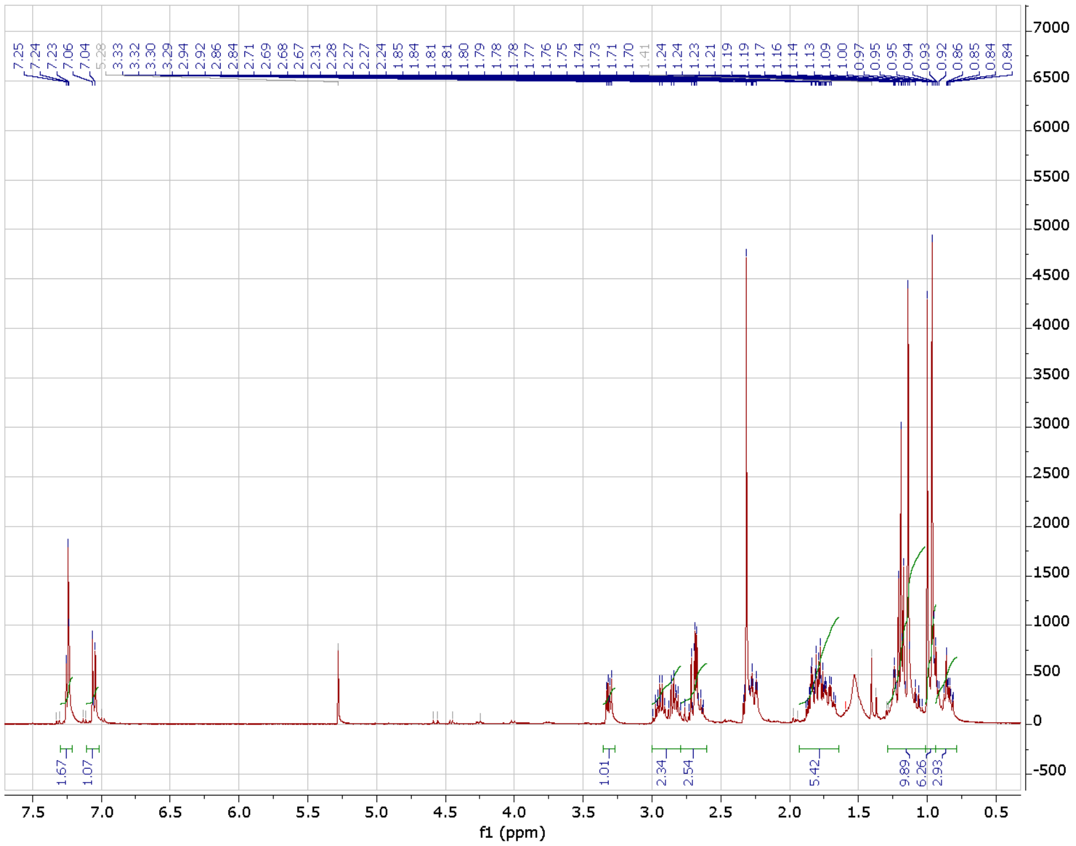

Figure S7. ^{1}H-NMR (500 MHz) Data for 7-oxo-8,11,13-cleistanthatrien-3-ol (**2**).

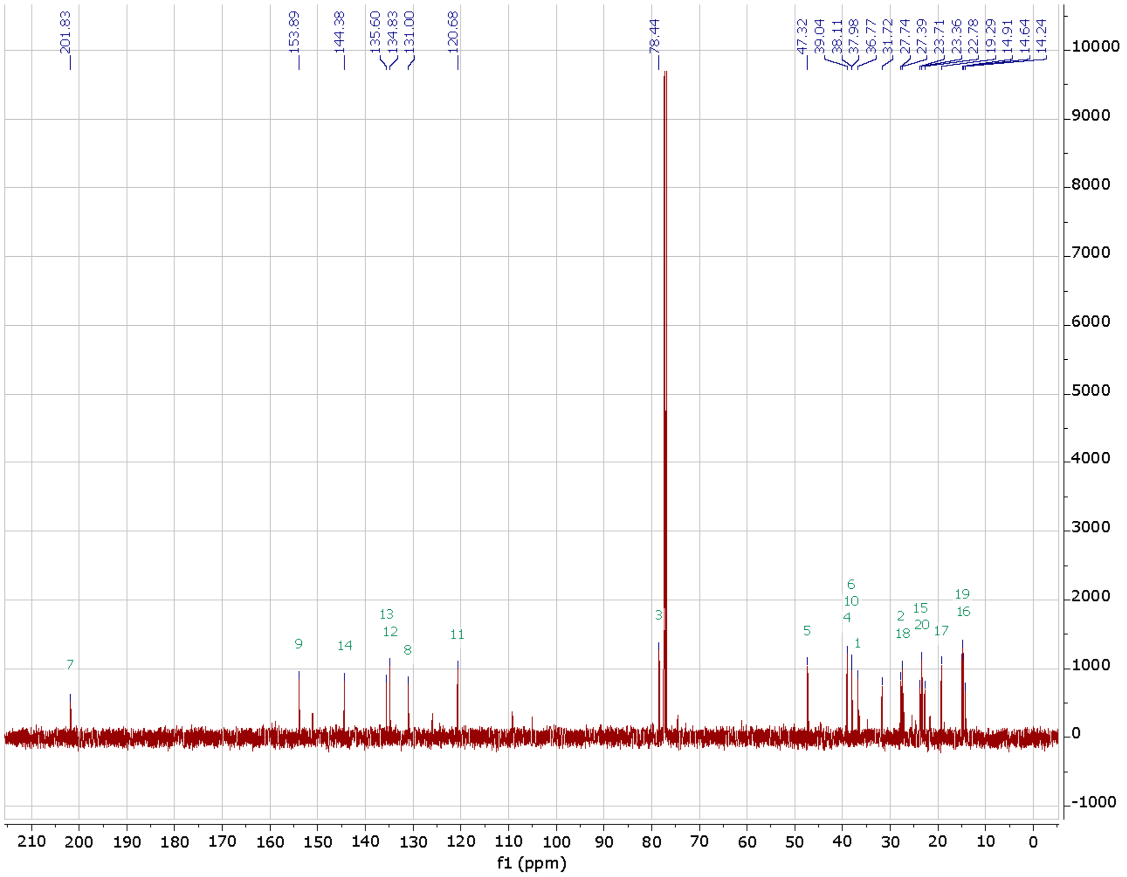

Figure S8. ^{13}C-NMR (125 MHz) Data for 7-oxo-8,11,13-cleistanthatrien-3-ol (**2**).

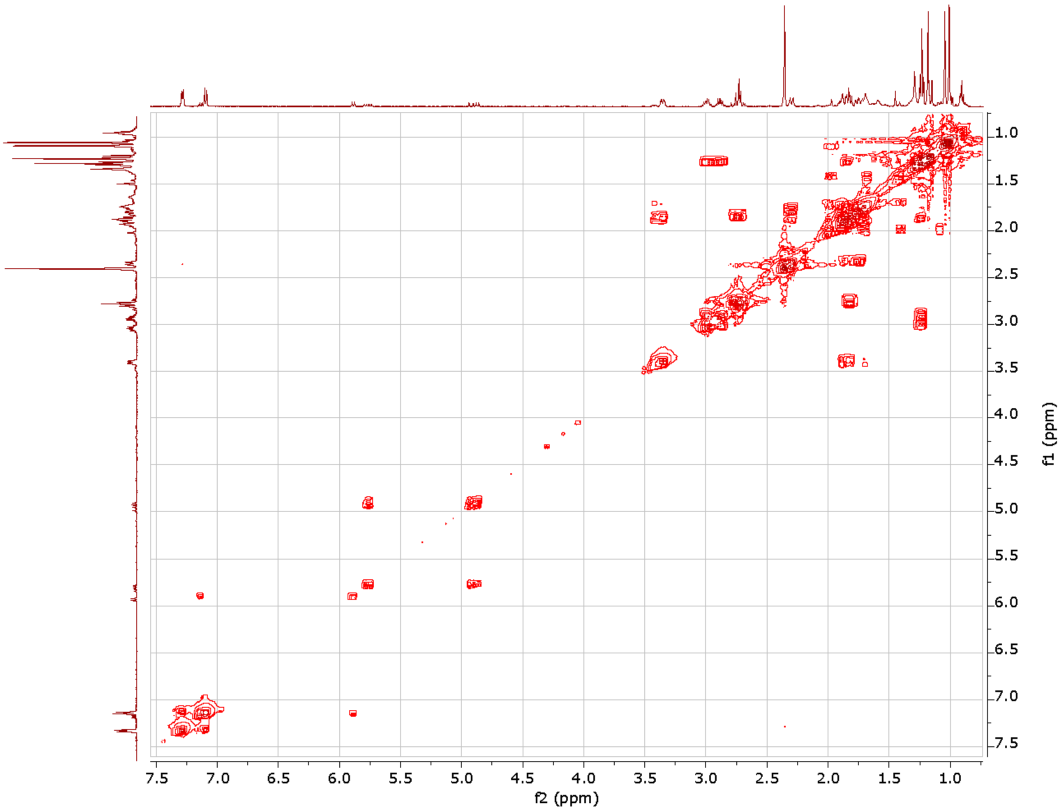

Figure S9. ^{1}H-^{1}H COSY (500 MHz) Data for 7-oxo-8,11,13-cleistanthatrien-3-ol (**2**).

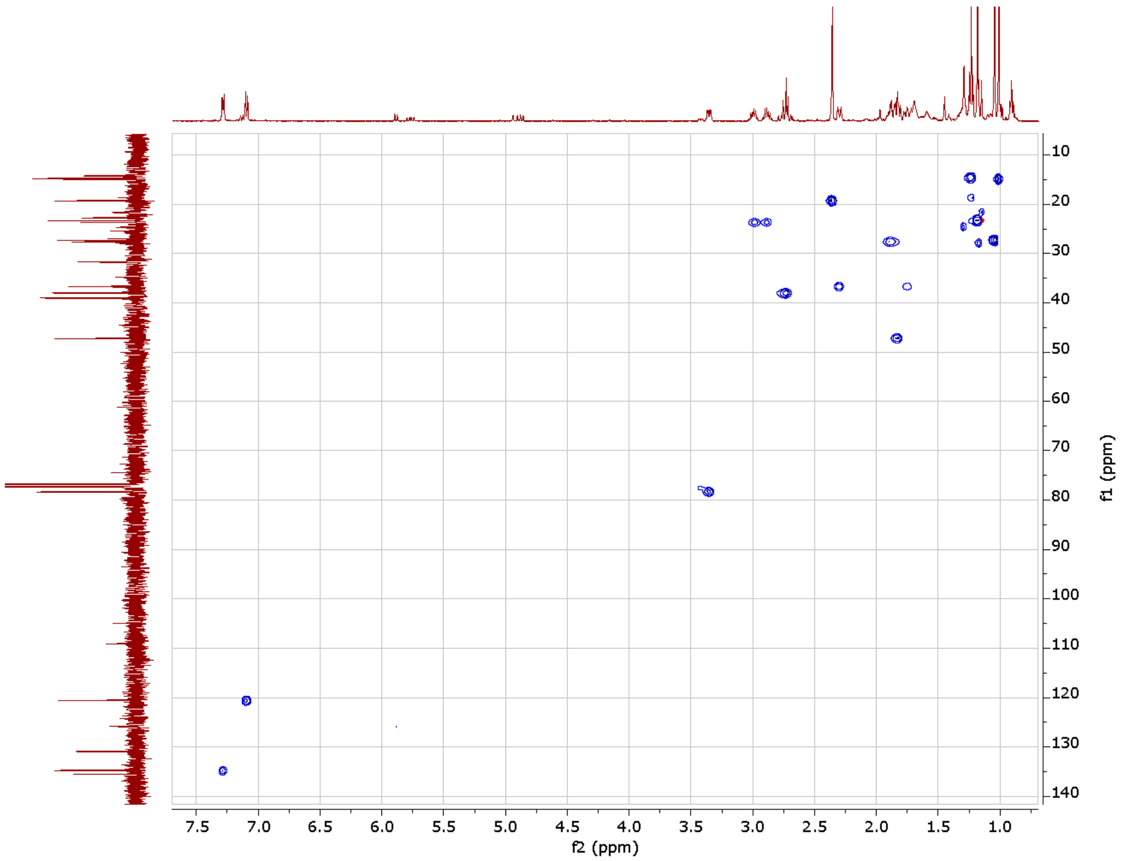

Figure S10. HSQC (500 MHz) Data for 7-oxo-8,11,13-cleistanthatrien-3-ol (**2**).

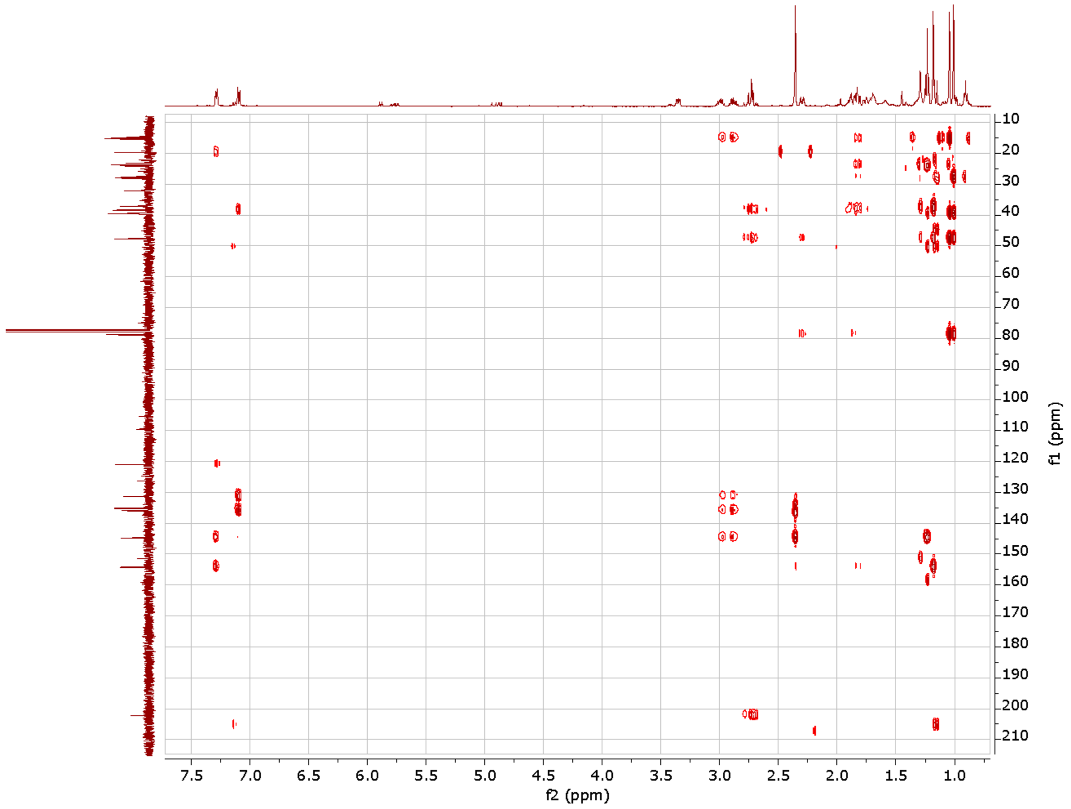

Figure S11. HMBC (500 MHz) Data for 7-oxo-8,11,13-cleistanthatrien-3-ol (**2**).

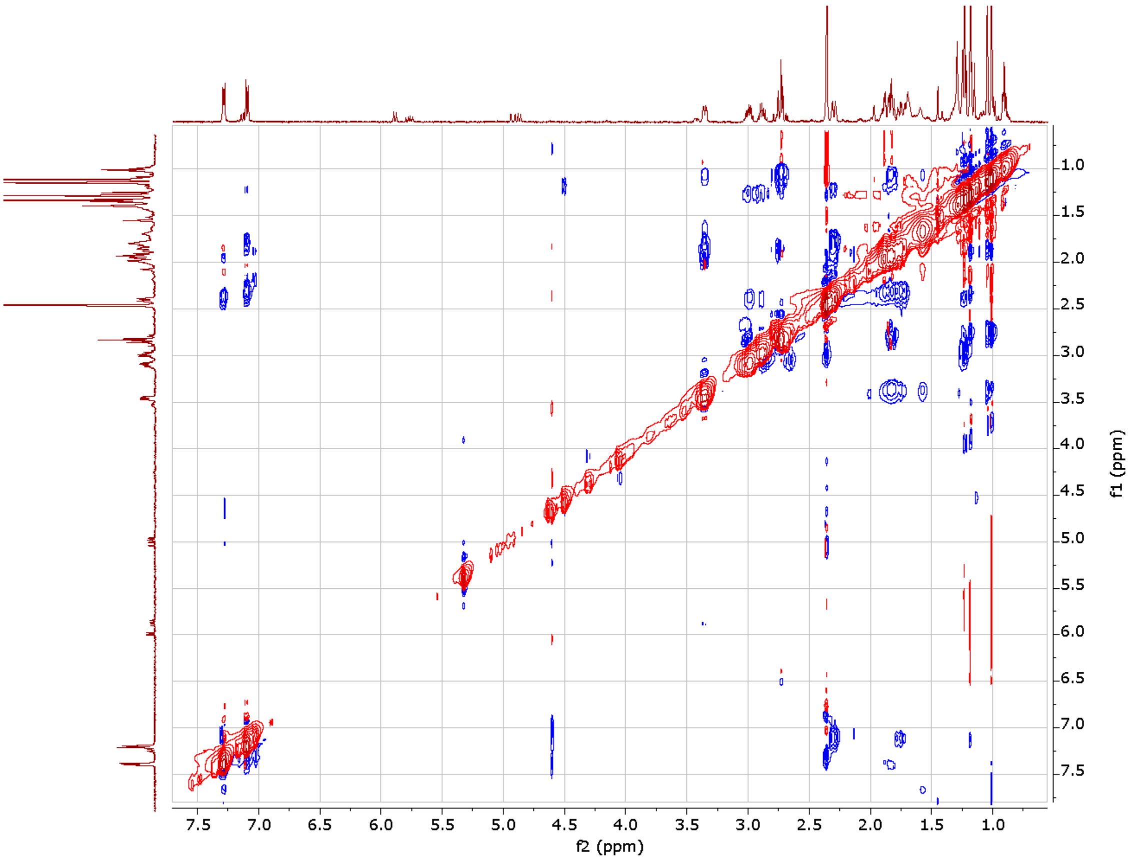

Figure S12. NOESY (500 MHz) Data for 7-oxo-8,11,13-cleistanthatrien-3-ol (**2**).

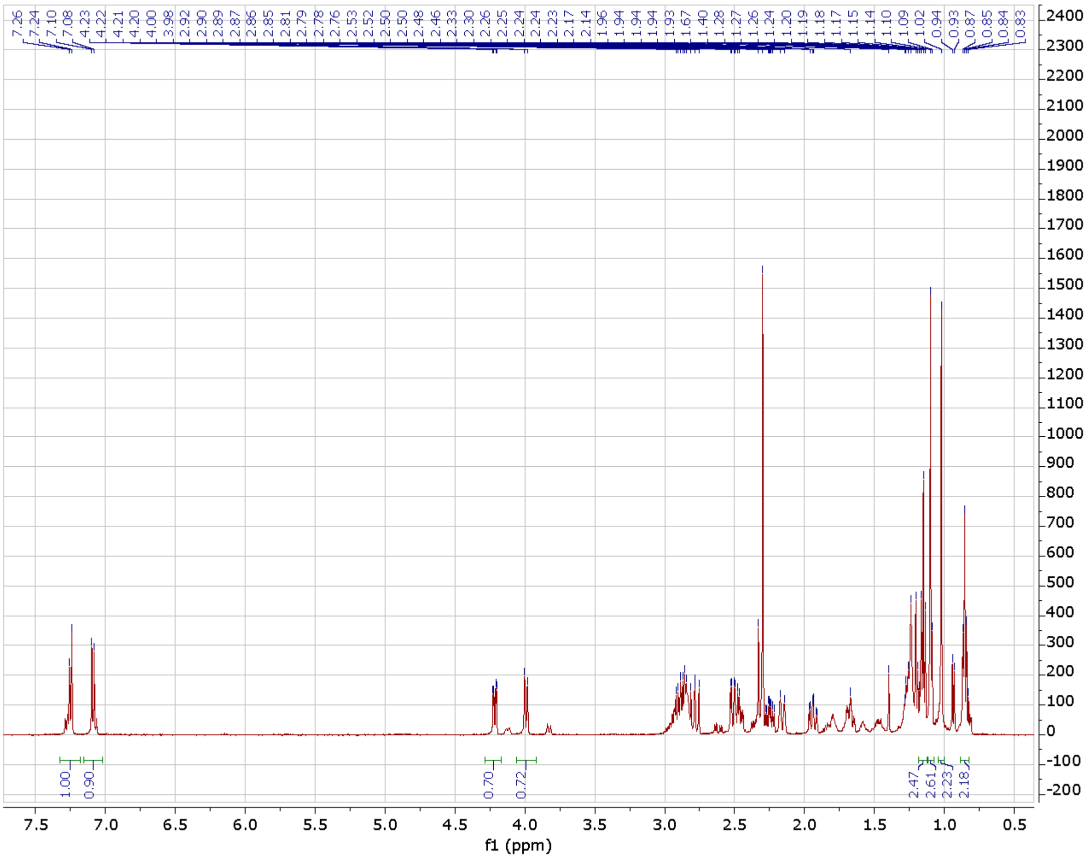

Figure S13. ^{1}H-NMR (500 MHz) Data for 3,20-epoxy-7-oxo-8,11,13-cleistanthatrien-3-ol (**3**).

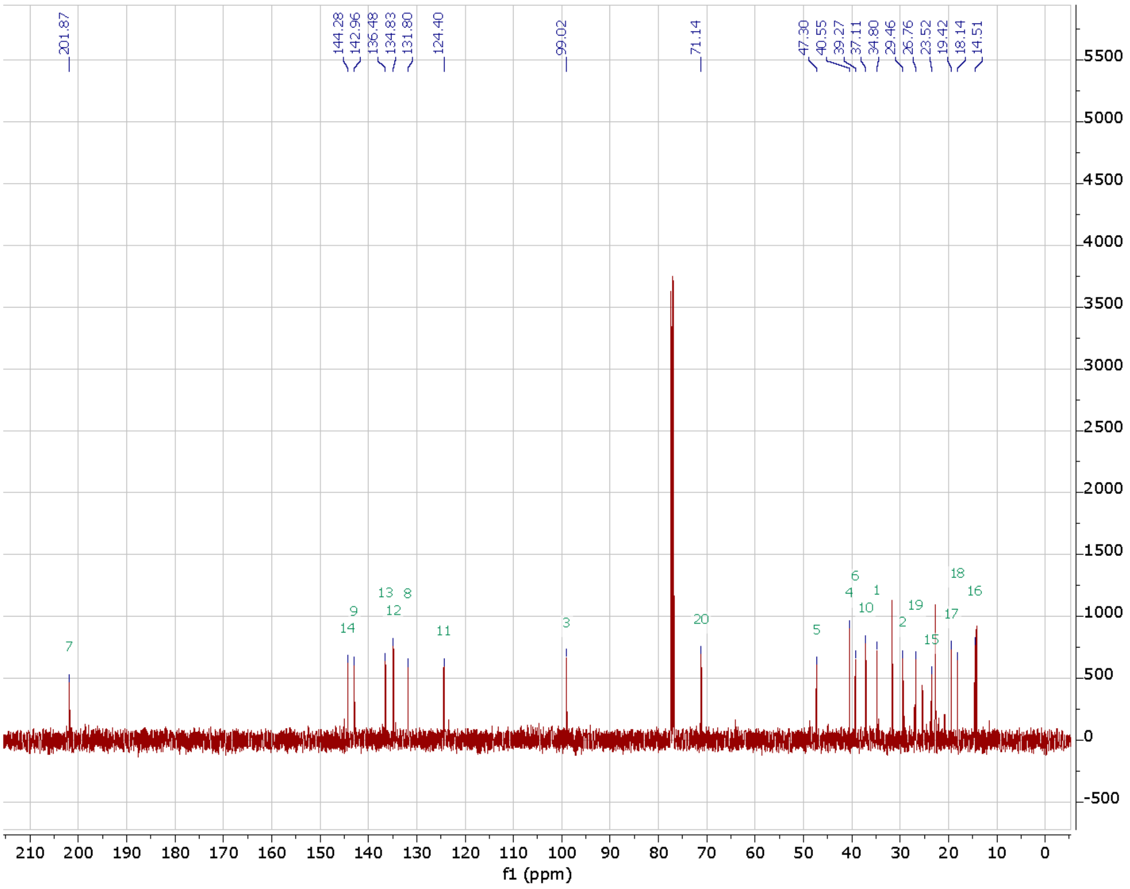

Figure S14. ^{13}C-NMR (125 MHz) Data for 3,20-epoxy-7-oxo-8,11,13-cleistanthatrien-3-ol (**3**).

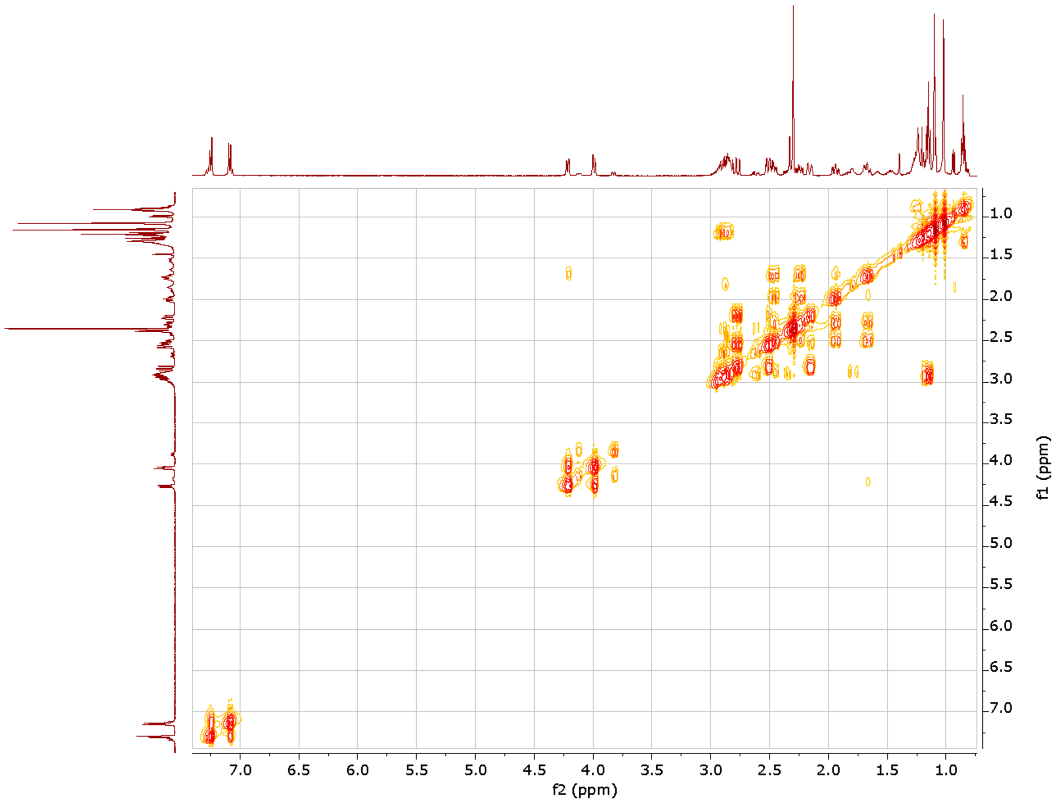

Figure S15. ^{1}H-^{1}H COSY (500 MHz) Data for 3,20-epoxy-7-oxo-8,11,13-cleistanthatrien-3-ol (**3**).

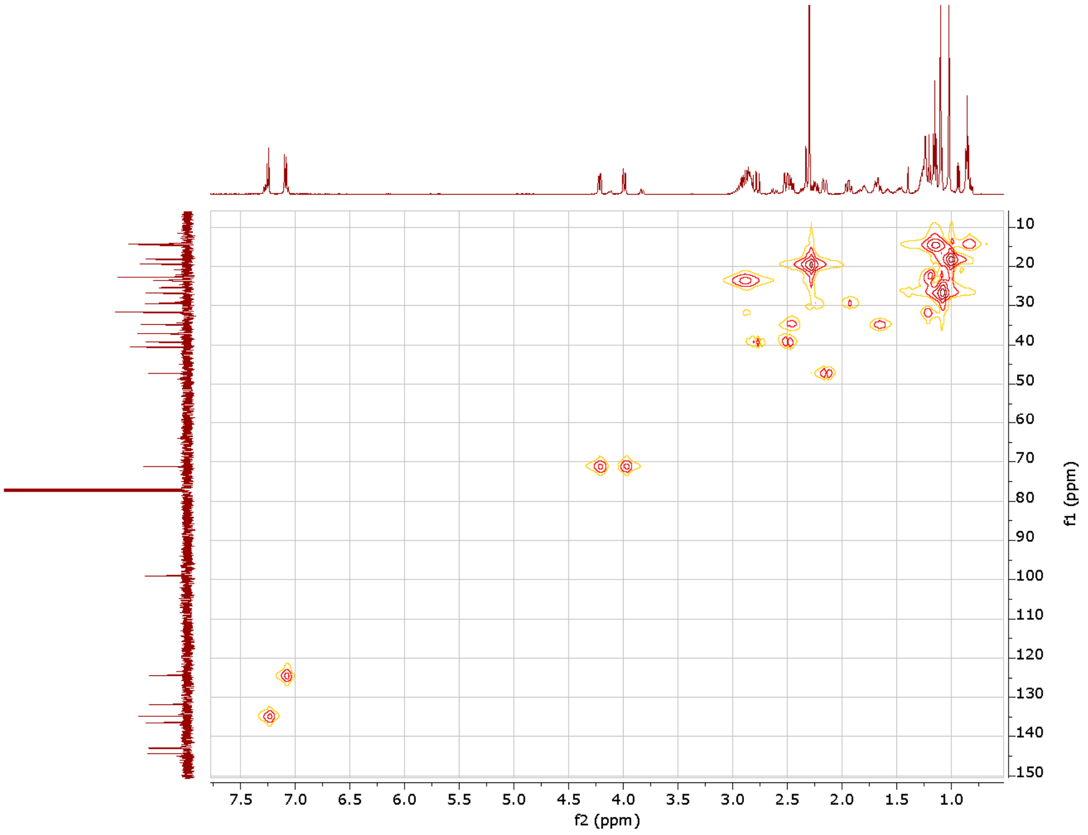

Figure S16. HSQC (500 MHz) Data for 3,20-epoxy-7-oxo-8,11,13-cleistanthatrien-3-ol (**3**).

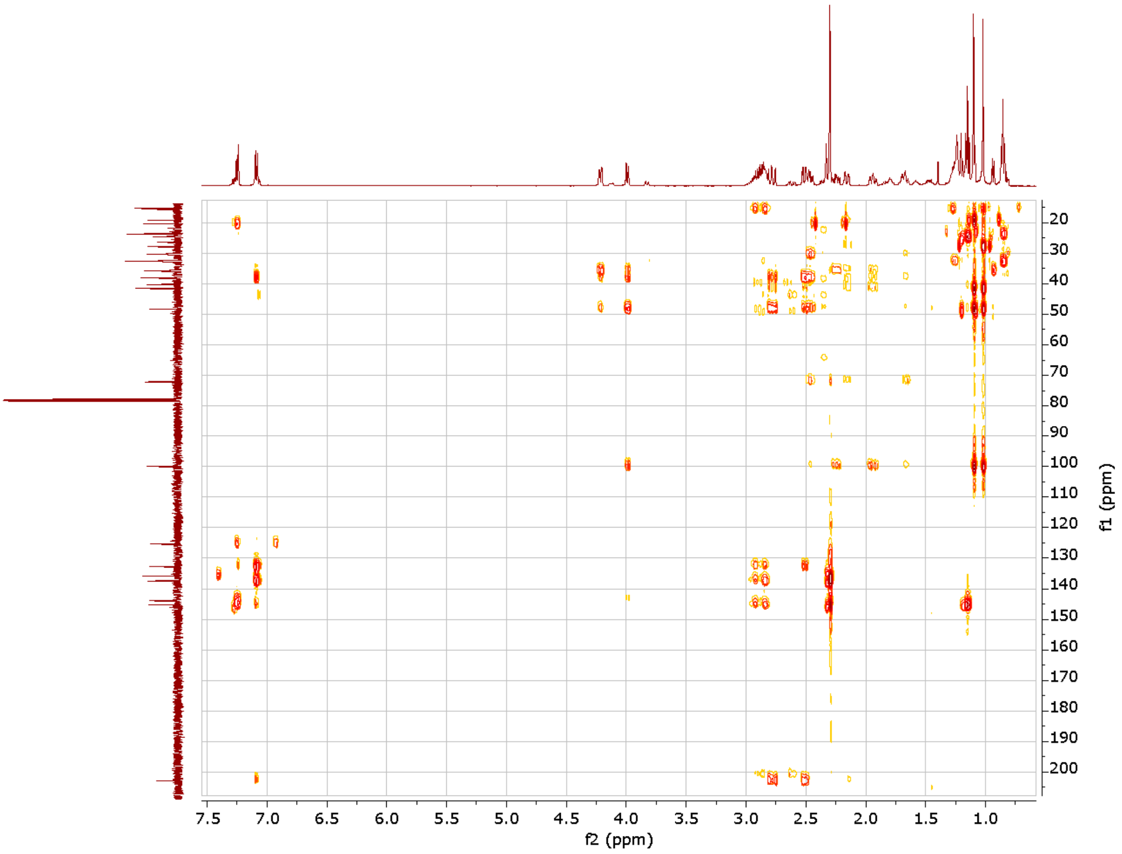

Figure S17. HMBC (500 MHz) Data for 3,20-epoxy-7-oxo-8,11,13-cleistanthatrien-3-ol (**3**).

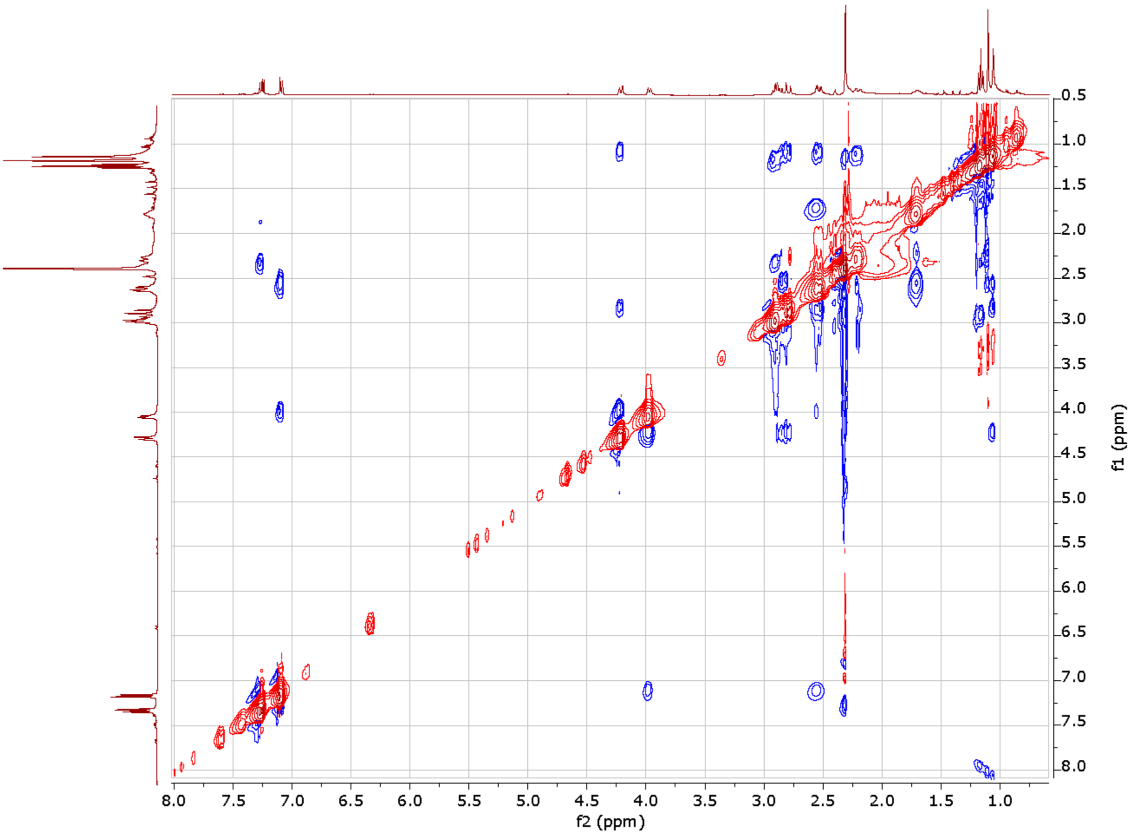

Figure S18. NOESY (500 MHz) Data for 3,20-epoxy-7-oxo-8,11,13-cleistanthatrien-3-ol (**3**).

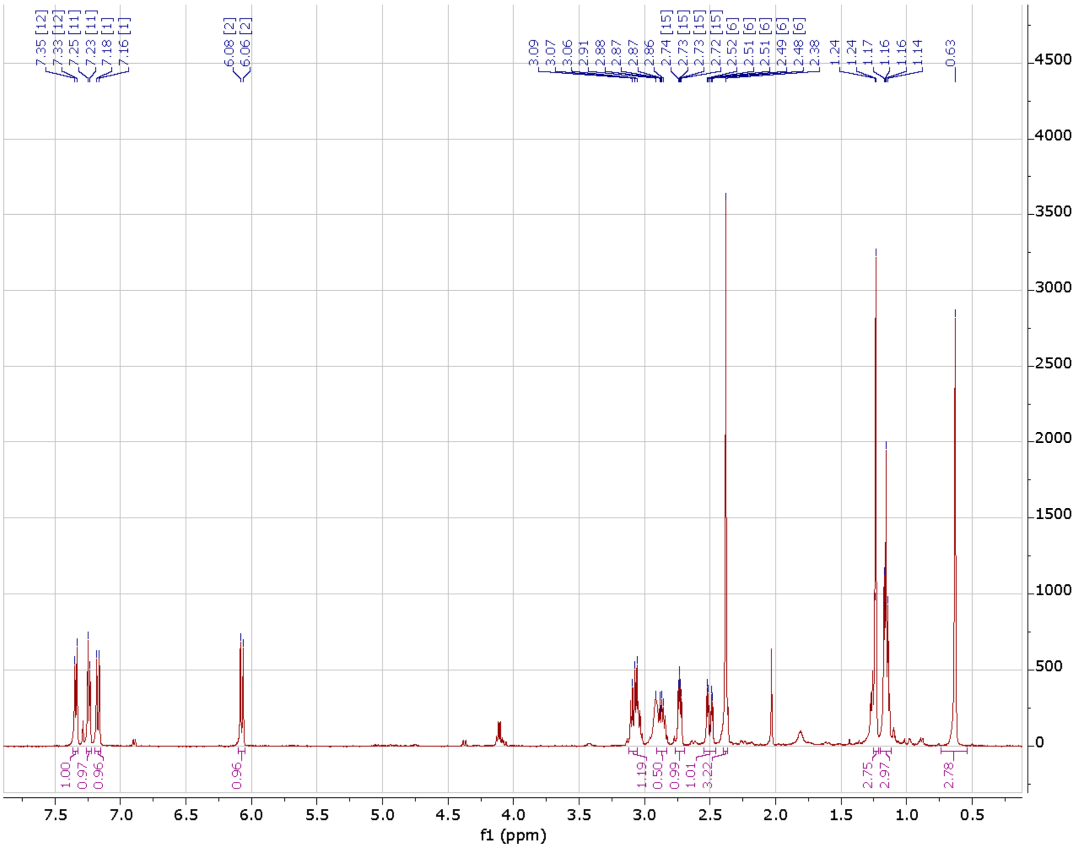

Figure S19. ^{1}H-NMR (500 MHz) Data for 20-nor-3,7-dioxo-1,8,11,13-cleistanthatetraen-10-ol (**4**).

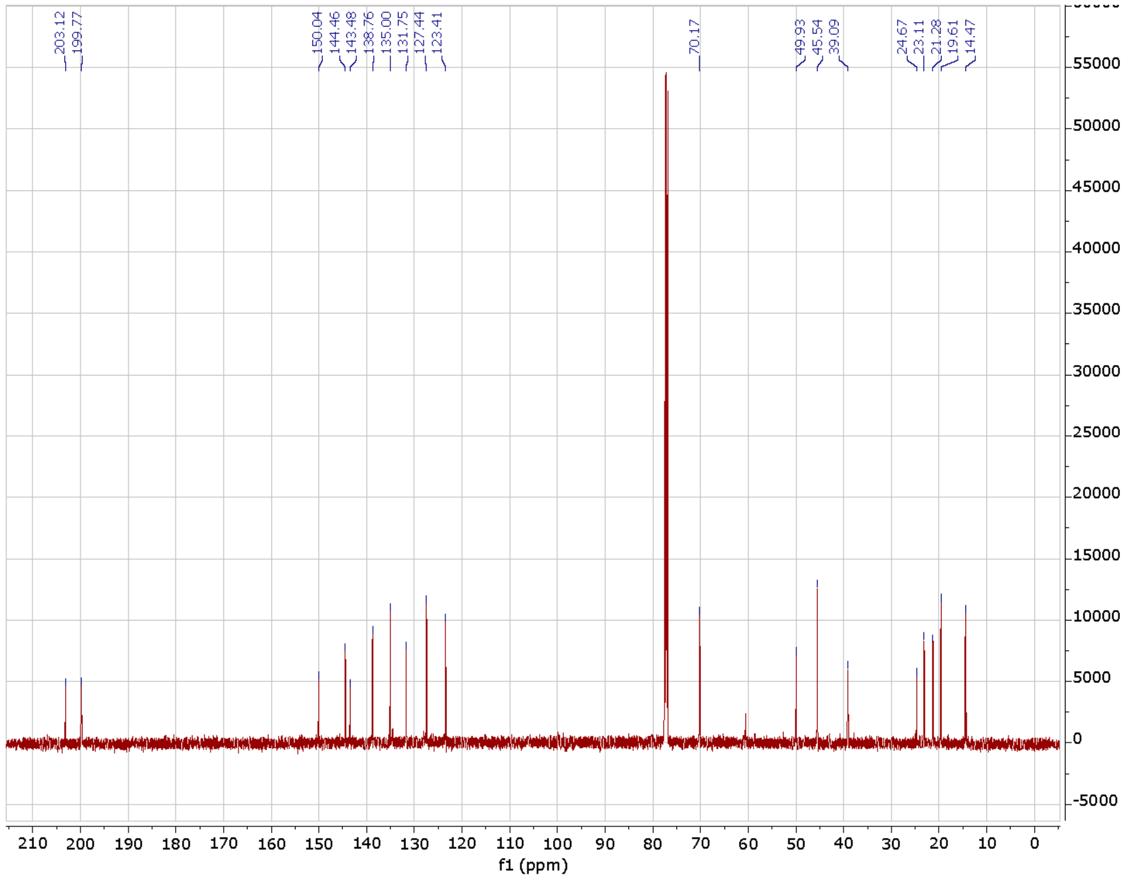

Figure S20. ^{13}C-NMR (125 MHz) Data for 20-nor-3,7-dioxo-1,8,11,13-cleistanthatetraen-10-ol (**4**).

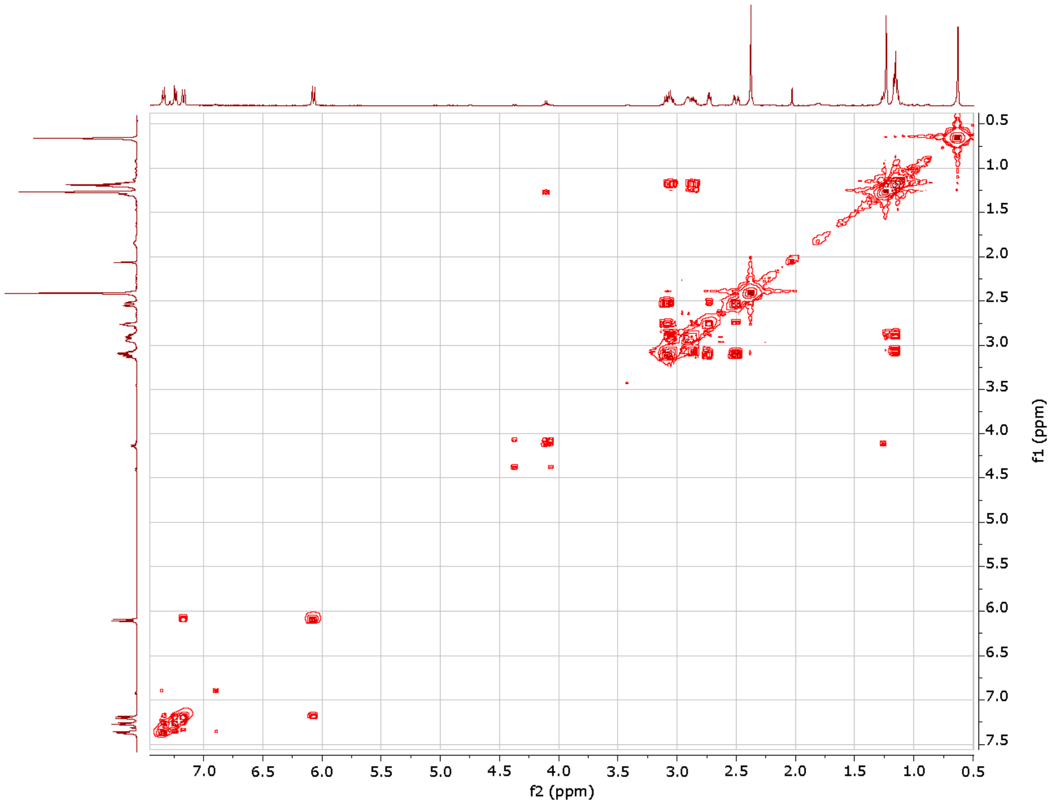

Figure S21. ^{1}H–^{1}H COSY (500 MHz) Data for 20-nor-3,7-dioxo-1,8,11,13-cleistanthatetraen-10-ol (**4**).

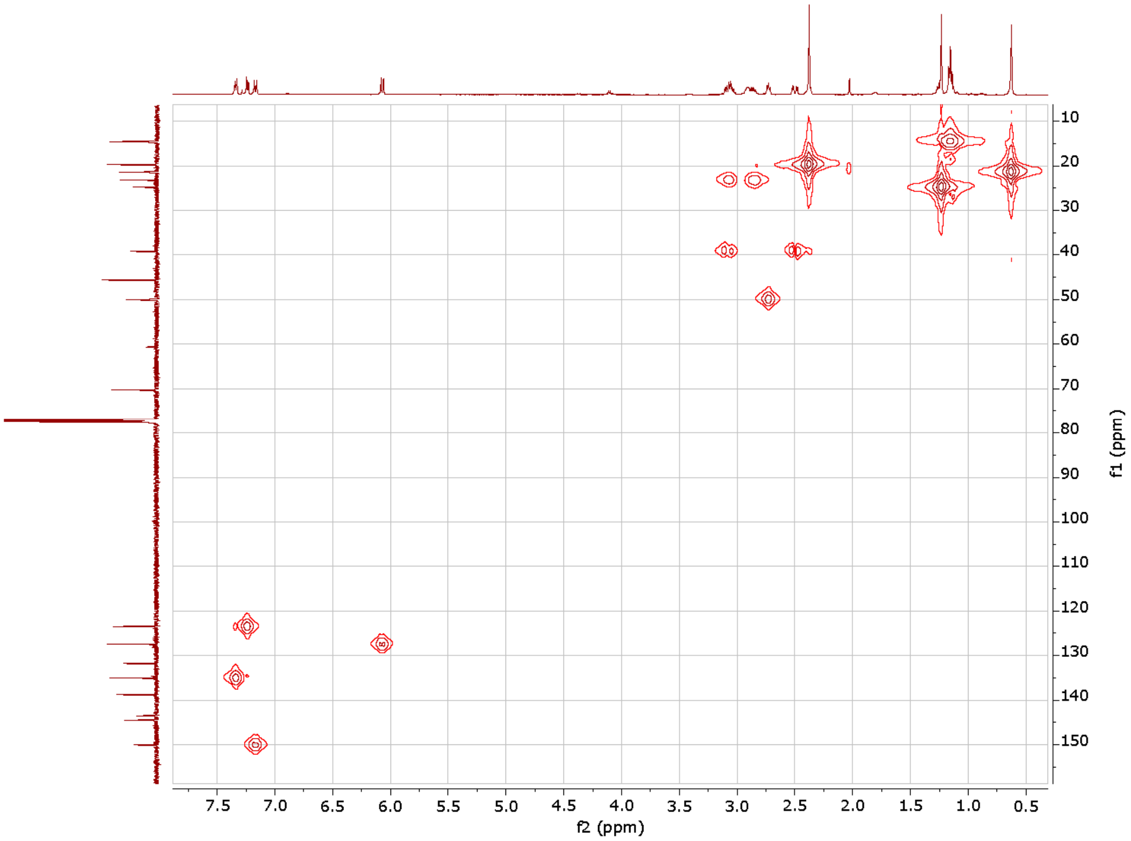

Figure S22. HSQC (500 MHz) Data for 20-nor-3,7-dioxo-1,8,11,13-cleistanthatetraen-10-ol (**4**).

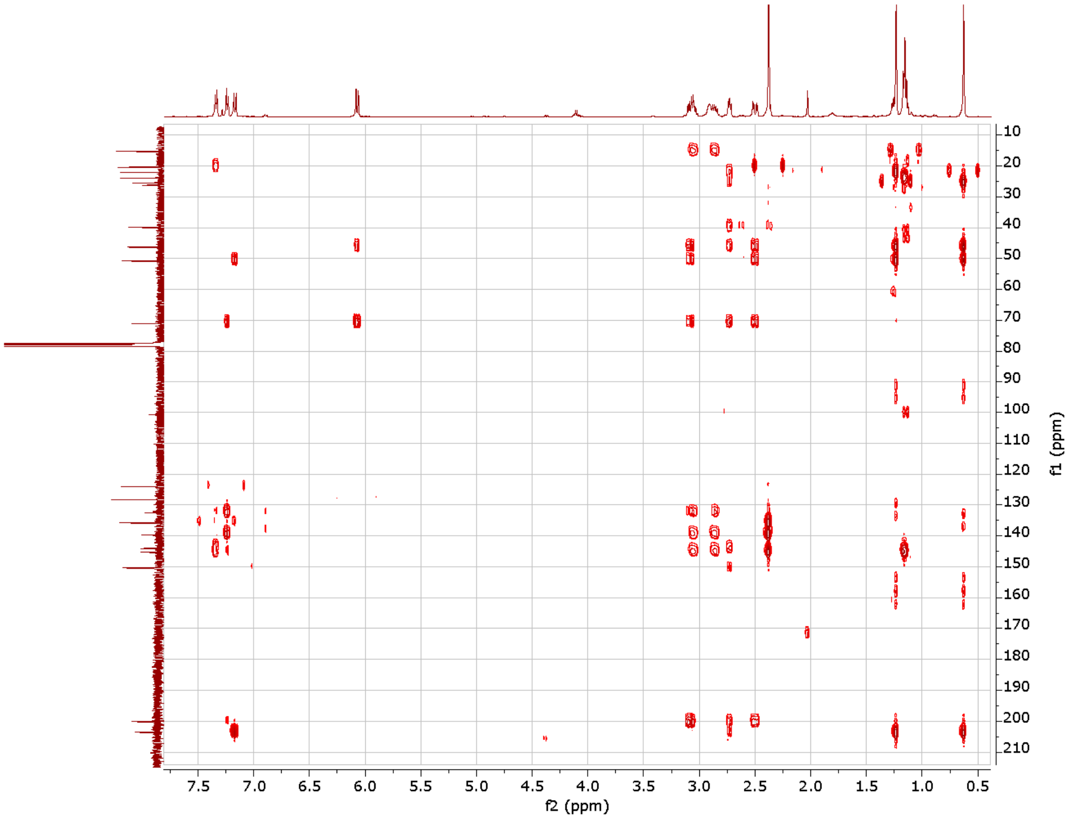

Figure S23. HMBC (500 MHz) Data for 20-nor-3,7-dioxo-1,8,11,13-cleistanthatetraen-10-ol (**4**).

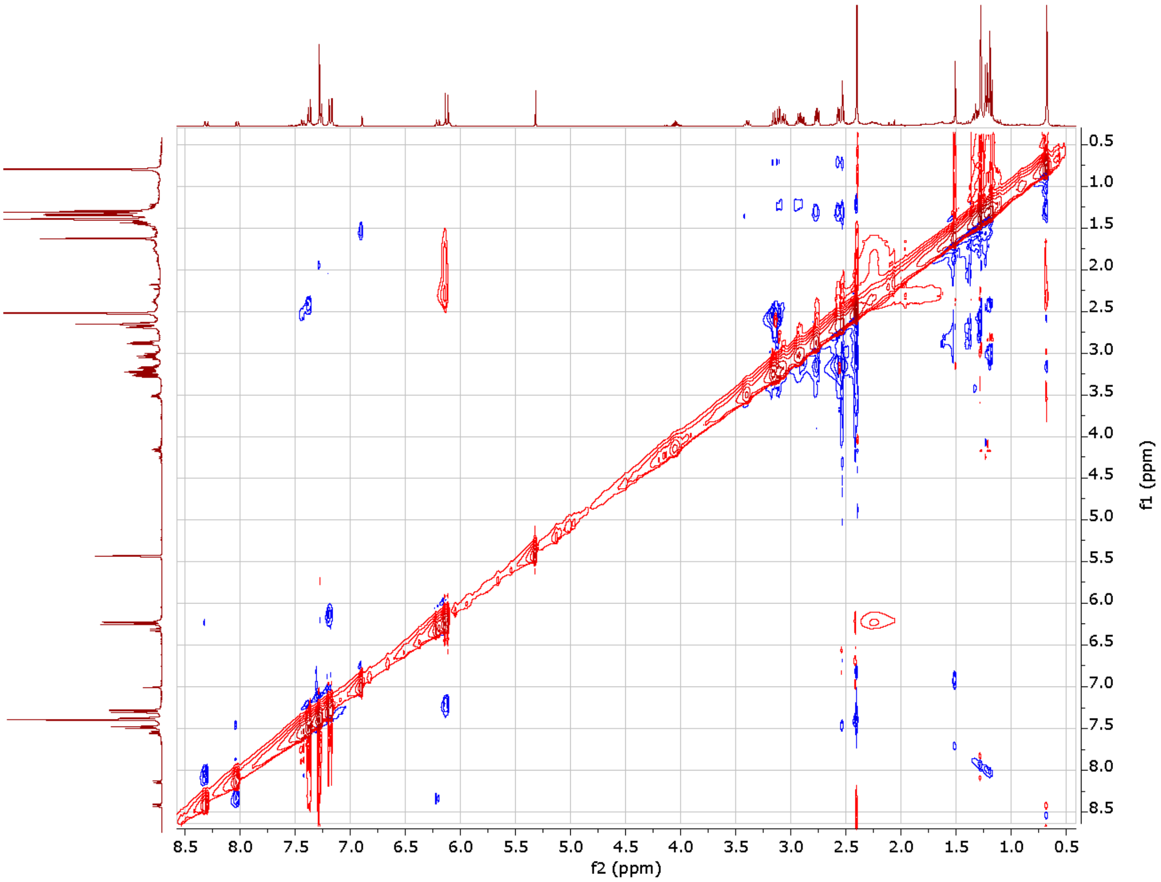

Figure S24. NOESY (500 MHz) Data for 20-nor-3,7-dioxo-1,8,11,13-cleistanthatetraen-10-ol (**4**).
